# Ligand-specific regulation of a binary enhancer code dictating cellular senescence

**DOI:** 10.1073/pnas.2506321122

**Published:** 2025-06-10

**Authors:** Thomas Suter, Meyer J. Friedman, Cagdas Tazearslan, Daria Merkurjev, Kenny Ohgi, Dario Meluzzi, Michael G. Rosenfeld, Yousin Suh

**Affiliations:** ^a^Cellular and Molecular Medicine, Department of Medicine, University of California San Diego, La Jolla, CA 92093; ^b^Department of Genetics, Albert Einstein College of Medicine, Bronx, NY 10461; ^c^Department of Obstetrics and Gynecology, Columbia University Irving Medical Center, New York, NY 10032; ^d^Department of Genetics and Development, Columbia University Irving Medical Center, New York, NY 10032

**Keywords:** cellular senescence, enhancers, SMAD2/3, NFI, p65

## Abstract

Cellular senescence, a major contributor to aging and age-related pathologies, is characterized by irreversible proliferative arrest and a disease-linked, proinflammatory profile known as the Senescence Associated Secretory Phenotype (SASP). A critical unanswered question is whether these properties are regulated by specific enhancer subsets, potentially licensing strategies that selectively block deleterious SASP components. Here, we identify two functionally distinct and independently regulated enhancer programs underlying senescence that are controlled by different TGF-β family ligands. Whereas Activin A stimulates recruitment of nuclear factor IA/C (NFIA/C) and SMAD2/3 transcription factors to an enhancer network that induces proliferation arrest, TGF-β2 promotes SMAD2/3-mediated suppression of a p65-dependent enhancer cohort driving the SASP. We have also uncovered reciprocal SMAD2/3-super-enhancer-regulated feedback loops that govern expression of the TGF-β2 (*TGFB2)* and Activin A (*INHBA*) transcription units, both of which are significantly up-regulated in replicative senescence. The characteristic enhancer usage and transcriptional landscape of high-passage senescent cells are sensitive to rapamycin treatment, discontinuation of which results in robust but selective senescent enhancer activation and exacerbation of the SASP. Collectively, this study uncovers separable enhancer programs and their key constituent transcription factors that contribute to the canonical features of cellular senescence, potentially informing the development of SASP-targeted therapies.

## Significance

Precise control of signal-dependent eukaryotic transcription, leading to specific alterations in gene expression, is mediated by the selective engagement of enhancer elements. We have identified two cohorts of regulatory enhancers that are responsive to distinct transforming growth factor-β (TGF-β) family ligands in replicative senescence via recruitment of SMAD2/3 and co-occupancy or exclusion of other pivotal transcription factors. These enhancer subsets drive gene expression programs associated with separate hallmarks of cellular senescence, namely permanent cell cycle arrest and a proinflammatory secretome. While both features of senescent cells can be beneficial in some contexts, the latter has been prominently implicated in age-related diseases. Thus, our findings suggest the possibility of constraining cellular senescence via interventions that selectively impact enhancer activity.

Cellular senescence is a complex stress response known to play a critical role in diverse physiological processes as well as age-related disorders (1–4). While highly heterogeneous, two hallmarks of cellular senescence are irreversible cell cycle arrest and the Senescence Associated Secretory Phenotype (SASP) (4–8), which occur in a cellular context- and signal-dependent manner. The permanent cessation of cellular proliferation is established and maintained by two main pathways, involving either p53 and p21 or p16^INK4a^ and pRB (2, 7, 9). The SASP is induced by factors downstream of p53 and p16 activation, such as p38 MAPK and NF-κB (10, 11), ultimately resulting in the release of a proinflammatory secretome, including cytokines, chemokines, and proteases, that alters the local tissue microenvironment but can also have distant effects.

Senescent cells increase with age and accumulate in various tissues in aging-associated diseases across mammalian species (4, 5, 8, 12–14). Copious evidence supports the deleterious role of SASP in pathological aging, as it reinforces senescence and spreads inflammation through autocrine and paracrine signaling (6, 15). Indeed, selective elimination of SASP-positive senescent cells can dramatically improve both life span and health span in mice (1–3, 9, 16–18). Yet, cellular senescence has important beneficial roles in biological processes such as development, tissue repair, and tumor suppression (7, 12, 15). The molecular mechanisms underlying cellular senescence remain poorly understood, posing a challenge in addressing whether its adverse aspect, the SASP, can be selectively targeted to promote healthy aging.

Specific molecular features of cellular senescence have been sequentially uncovered, which include numerous, robust transcriptional changes in gene expression (6, 15, 19, 20). In addition, alterations in histone modifications, chromosomal architecture/interaction networks (21–24), DNA methylation (25), lamina-associated domains (23, 26, 27), and transposable element activity (28) have been observed. However, there remains a pressing need for a clear hypothesis that can explain the underlying transcriptional and epigenomic mechanisms that dictate cellular senescence and distinguish the two distinct outcomes/aspects with which it is associated. Transcriptional enhancers serve as major points of integration of intra- and extracellular signals associated with development, homeostasis, and disease (29–34). Moreover, signals such as inflammation and cellular or DNA damage can activate enhancers not previously functioning during development (35–38). Thus, we have sought to better understand the role of enhancers as determinants of senescence programs.

Here, we provide evidence that the irreversible growth arrest and the SASP aspects of cellular senescence are independently implemented by two gained enhancer cohorts, which are selectively activated by NFIA/C and NF-κB, respectively. In turn, both programs are regulated by SMAD2/3 via TGF-β signaling. Our results also reveal that expression of the TGF-β ligands Activin A and TGF-β2, which we implicate in the distinct senescent enhancer programs, is under super-enhancer control. The activation of these super-enhancers in replicative senescence mediates previously uncharacterized SMAD2/3-dependent regulatory feedback loops. While TGF-β ligands are well-established components of the SASP, their individual contributions to features of senescence, especially those governed by enhancer-mediated transcriptional regulation, are largely unknown. We show that Activin A stimulates the enhancer program associated with proliferation arrest through recruitment of NFIA/C, up-regulating a large collection of genes that includes *INHBA*, encoding the Activin A monomer that assembles into a functional homodimer, and *TGFB2*. In contrast, TGF-β2 functions to suppress the NF-κB-activated SASP enhancer program but also up-regulates *INHBA* in addition to its own transcription unit. These observations establish a mechanistic rationale for further exploration of strategies, such as a combined treatment of TGF-β2 and the natural Activin A antagonist Inhibin A, that could potentially preserve the functional capacity of certain aging tissues by selectively blocking the enhancer cohort that drives the deleterious SASP program of cellular senescence.

## Results

### Enhancers in Replicative Senescence.

**Fig. 1.**
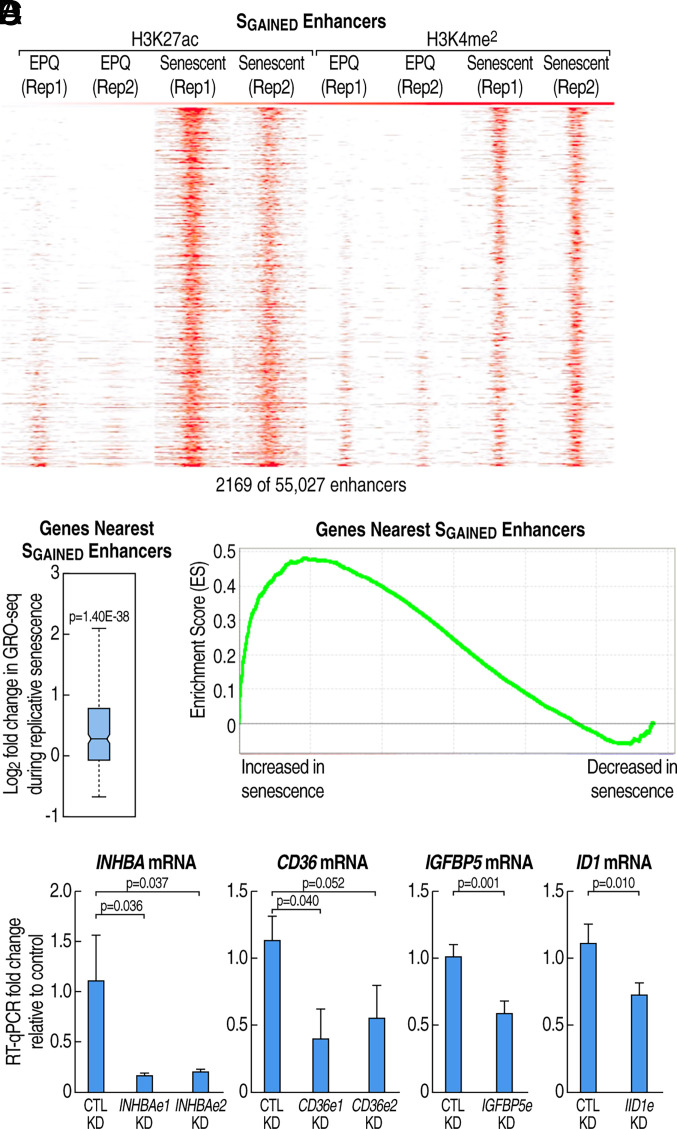
Gained enhancers regulate the replicative senescence-induced transcription program. (*A*) Heatmap of H3K27ac and H3K4me^2^ ChIP-seq enrichment in EPQ and senescent cells at 2169 S_GAINED_ enhancers. Each column represents a −/+ 3 kb window centered on the enhancer midpoint. Enhancers were sorted based on the average fold change of HOMER-determined peak scores in replicative senescence. (*B* and *C*) Analysis of transcriptional profile of genes above expression threshold located nearest to 2169 S_GAINED_ enhancers. (*B*) Box plot of log_2_ fold change in expression of these genes during senescence (*P* values for all box plots determined by two-tailed z-tests; see *Materials and Methods* for additional information regarding box plots and significance testing). (*C*) Gene Set Enrichment Analysis (GSEA) plot of enrichment score (45) for aforementioned gene set over background dataset of 14,493 genes detectibly expressed in BJ fibroblasts. Expressed genes on the x-axis were ranked by average fold change in replicative senescence based on combined RNA-seq and GRO-seq data (*SI Appendix*, *Extended Methods*). (*D*) RT-qPCR detection of fold change in senescence-induced mRNA transcripts upon shRNA-targeting of indicated S_GAINED_ enhancers (see *Materials and Methods* for details about eRNA knockdown via shRNA). Error bars represent SD (n ≥ 3 biological replicates). *P* values were determined by two-tailed *t* tests.

In order to examine the gene regulatory landscape associated with replicative senescence, we first characterized the difference in enhancer profiles between late-passage senescent and early-passage quiescent (EPQ) BJ fibroblasts. Senescence was induced by repeated passaging (see *Materials and Methods* for culturing conditions), while EPQ cells were used as controls in order to minimize the impact of cell cycle effects in our comparisons. To identify enhancers, we performed Chromatin Immunoprecipitation followed by sequencing (ChIP-seq) in duplicate for H3K4me^2^ and H3K27ac histone modifications in EPQ and senescent cells, focusing on nonpromoter regions containing peaks of both marks. This approach yielded 2169 gained enhancers (Senescence Gained: S_GAINED_) in replicative senescence, based on a twofold increase in H3K27ac and a 1.5-fold increase in H3K4me^2^ signal intensity (Fig. 1*A*), which was replicated in three separate experiments. GWAS/PheWAS analysis overlapping SNPs located in S_GAINED_ enhancers with a database of age-related traits showed significant enrichment for many SNP-linked traits (39, 40), including coronary artery disease, rheumatic heart disease, multiple sclerosis, and chronic lymphocytic leukemia, consistent with a role for these enhancers in human aging-associated pathology (*SI Appendix*, Fig. S1*A*). Conversely, 1565 enhancers were reproducibly lost during senescence (Senescence Lost: S_LOST_), based on joint decreases in H3K27ac and H3K4me^2^ of twofold and 1.5-fold, respectively. As many as 51,293 enhancers were unaltered in senescent cells using these thresholds. We interrogated the same enhancer marks in the context of replicative and oncogene-induced senescence (OIS) in IMR90 embryonic lung fibroblasts. IMR90 cells reached replicative senescence through repeated passaging, and OIS was induced by exposure of early passage cells to virally expressed H-Ras. Both S_GAINED_ and S_LOST_ enhancers, as defined in BJ fibroblasts, were conserved in IMR90 cell models of replicative senescence and OIS (*SI Appendix*, Fig. S1 *B* and *C*), supporting the generality of the findings. These initial observations are consistent with reported dynamic changes in enhancer usage in aging tissues (41) and senescent cells (42–44).

We next investigated the potential function(s) of the gained enhancers by integrating our data on transcriptional changes in replicative senescence from a parallel study, involving Global Run-On sequencing (GRO-seq) and RNA sequencing (RNA-seq) of early passage and senescent BJ fibroblasts (GEO accession GSE146585) (46). Coding genes near S_GAINED_ enhancers displayed increased transcription during replicative senescence (Fig. 1 *B* and *C*). Furthermore, knockdown of six S_GAINED_ eRNAs, each with multiple shRNAs, resulted in a significant reduction of the nearby target genes (Fig. 1*D*), consistent with the conclusion that S_GAINED_ enhancers serve a regulatory function that shapes the transcriptome during the senescence process. In contrast to S_GAINED_ enhancers, the promoters of genes that were significantly up-regulated during replicative senescence (S_GAINED_) did not feature increased H3K27ac or H3K4me^2^ (*SI Appendix*, Fig. S1*D*; see *SI Appendix*, *Extended Methods* for gene expression set and subset criteria).

### Effects of Rapamycin Treatment and Its Cessation on Replicative Senescence Enhancers and Transcription.

**Fig. 2.**
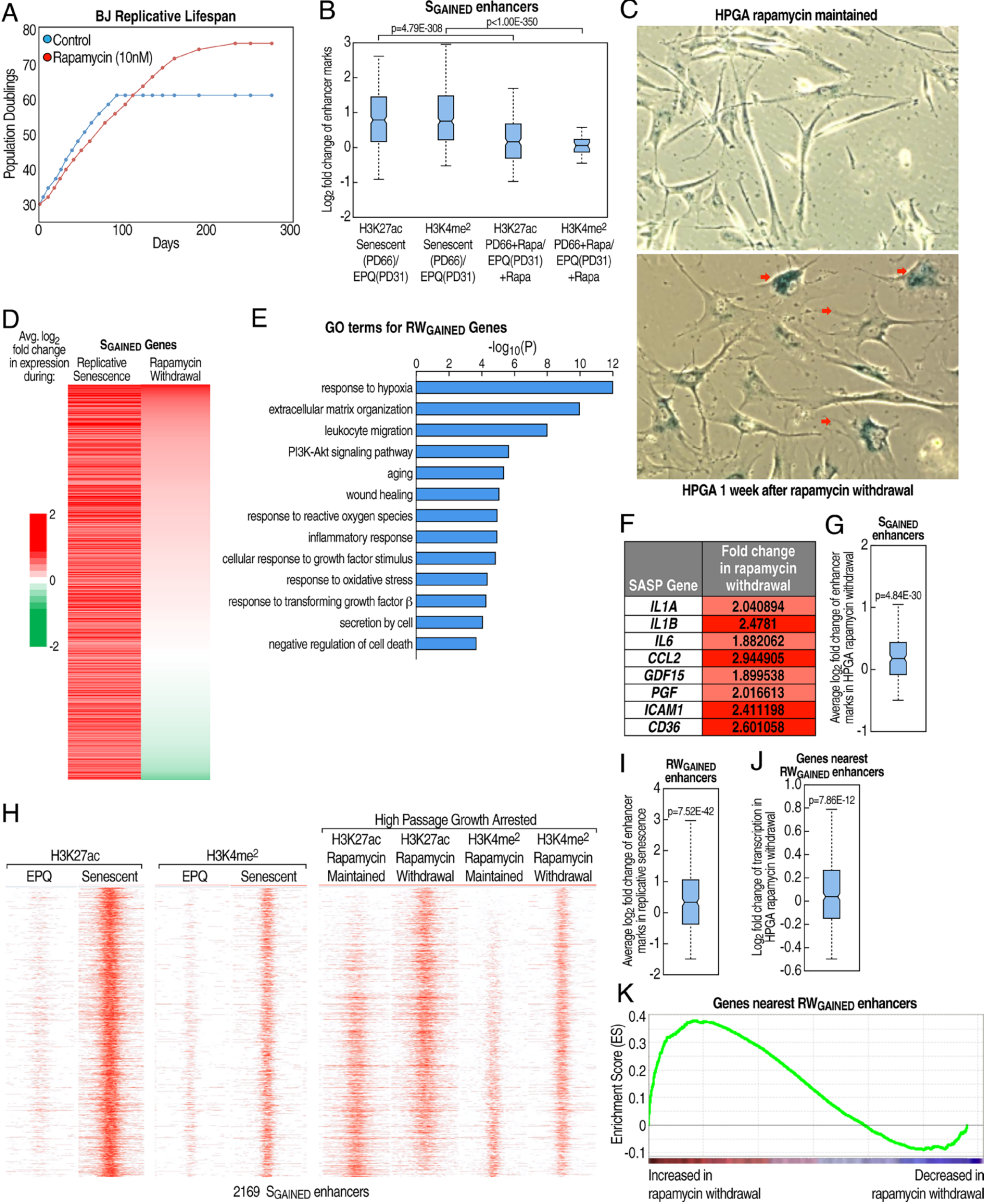
Withdrawal of HPGA cells from rapamycin maintenance distinguishes and accentuates the SASP program of replicative senescence. (*A*) Growth curves for BJ fibroblasts treated with 10 nM rapamycin or vehicle control. (*B*) Box plots of fold change in enhancer marks comparing senescent and EPQ cells in the presence or absence of 10 nM rapamycin. Fold change is based on the log_2_ ratio of peak scores for the indicated conditions. S_GAINED_ enhancers were redefined for this panel alone by excluding the replicates used to generate these box plots. (*C*) SA-β-gal staining of HPGA BJ fibroblasts maintained in 10 nM rapamycin (RM) or withdrawn from rapamycin treatment for 1 wk following extended administration (RW). HPGA RW but not RM cultures featured enlarged cells with a flattened morphology and positive SA-β-gal staining of varying intensities (red arrows). (*D*) Heatmap of average log_2_ fold change in transcription of 639 S_GAINED_ genes during replicative senescence or rapamycin withdrawal, sorted based on the latter. (*E*) Selected GO-terms from Metascape analysis of 252 RW_GAINED_ genes. (*F*) Table of average fold change in transcription of selected SASP genes in rapamycin-maintained HPGA BJ fibroblasts following rapamycin withdrawal. (*G*) Box plot of fold change in enhancer histone marks for rapamycin-withdrawn HPGA fibroblasts at 2169 S_GAINED_ enhancers. Averaged fold change in H3K27ac and H3K4me^2^ peak scores upon rapamycin withdrawal is plotted. (*H*) Heatmap of H3K27ac and H3K4me^2^ tag density at 2169 S_GAINED_ enhancers in EPQ, senescent, HPGA rapamycin maintained, and HPGA rapamycin withdrawal conditions. Enhancers were sorted based on the fold change of H3K27ac in rapamycin withdrawal. Each column displays a −/+ 3 kb window from the enhancer center. (*I*) Box plot of fold change in enhancer peaks during replicative senescence at 1512 RW_GAINED_ enhancers. Averaged fold change in H3K27ac and H3K4me^2^ peak scores in replicative senescence is plotted. (*J* and *K*) Analysis of transcriptional profile of genes above expression threshold located nearest to 1512 RW_GAINED_ enhancers. (*J*) Box plot of fold change in gene set for rapamycin-withdrawn HPGA cells. (*K*) GSEA plot of enrichment score of aforementioned gene set over a background dataset of 14,493 genes in BJ fibroblasts exceeding a minimum expression threshold. Expressed genes on the x-axis were ranked by fold change in HPGA rapamycin-withdrawn cells.

To further explore the role of the S_GAINED_ enhancer program in senescent phenotypes, we treated BJ fibroblasts with rapamycin, a potent inhibitor of mTOR that promotes life span in various model systems (47), including mice (48–51), and delays the onset of replicative senescence (52, 53). Long-term treatment of BJ fibroblasts with rapamycin (10 nM) reproducibly resulted in a ~25% increase in the number of population doublings before proliferative arrest (Fig. 2*A*) and significantly fewer gained enhancers in replicative senescence (Fig. 2*B* and *SI Appendix*, Fig. S2*A*). Even after reaching proliferative arrest following many (70+) population doublings, High Passage Growth-Arrested (HPGA) cells maintained in rapamycin retained the morphology of early passage cells and were negative for senescence-associated beta-galactosidase (SA-β-gal) staining (Fig. 2*C* and *SI Appendix*, Fig. S2*B*). Because rapamycin treatment seemed to prevent certain features of senescence even in proliferatively arrested cells, we investigated whether these changes were stable upon rapamycin withdrawal. HPGA cells withdrawn from rapamycin treatment reverted to a senescent-like state just 2 wk after drug removal, with a dramatic onset of senescent morphology and increased SA-β-gal staining (Fig. 2*C* and *SI Appendix*, Fig. S2*B*). RNA-seq of HPGA cells withdrawn from rapamycin versus those maintained in rapamycin showed a clear correlation between the genes induced upon rapamycin withdrawal (RW_GAINED_) and S_GAINED_ genes (Fig. 2*D* and *SI Appendix*, Fig. S2 *C* and *D*). Strikingly, GO analysis of the RW_GAINED_ genes revealed enrichment of categories associated with inflammatory and secretory pathways, suggesting activation of an enhancer cohort specifically connected to the SASP senescence program (Fig. 2*E*). Consistently, the RW_GAINED_ genes included many hallmark SASP genes, such as *IL1A*, *IL1B*, *IL6*, *CCL2*, and *ICAM1* (Fig. 2*F*). ChIP-seq of H3K27ac and H3K4me^2^ demonstrated a pronounced increase in both marks at S_GAINED_ enhancers in HPGA rapamycin-withdrawn versus rapamycin-maintained BJ fibroblasts (Fig. 2 *G* and *H*). Moreover, examination of 1512 RW_GAINED_ enhancers, defined in the same fashion as S_GAINED_ enhancers, showed that they were marked for activation in replicative senescence (Fig. 2*I*) and that their nearest expressed genes were up-regulated and enriched in the rapamycin withdrawal-induced transcriptional program (Fig. 2 *J* and *K*). These results indicate that although continued treatment of cells with rapamycin does not prevent eventual proliferative arrest, it effectively blocks SASP manifestation due to a failure to activate the S_GAINED_, SASP-specific enhancer program in its presence; however, rapamycin withdrawal from HPGA cells causes a rapid onset of the SASP, mediated by activation of a conserved enhancer cohort and induction of its associated gene repertoire.

Enhancer programs are associated with changes in epigenomic landscape and 3D chromosomal architecture (30, 33, 34), both of which have been implicated in senescence (21–24). To assess whether alterations in global genomic structure correlated with enhancer function and transcriptional profiles in senescence, we performed in situ High-throughput Chromosome Conformation Capture (Hi-C) in EPQ and senescent BJ cells. We observed highly significant changes in A/B compartmentalization during replicative senescence, which were robustly conserved in rapamycin-withdrawn cells and reversed upon p65 inhibition or by rapamycin treatment (*SI Appendix*, Fig. S2 *E*–*H*). However, the compartmental shifts did not significantly correlate with directional changes in mRNA levels in senescence (*SI Appendix*, Fig. S2 *I*–*K*) or with enhancer transcriptional programs (*SI Appendix*, Fig. S2 *L* and *M*). These results suggest that enhancer-driven transcriptional changes in gene expression that characterize replicative senescence do not require large-scale chromosomal structural reorganization, but the contributions of TAD-level remodeling, as has been previously documented in replicatively senescent cells (54), and sub-TAD alterations cannot be excluded. Notably, we detected a significant amount of clustering within the S_GAINED_ gene set based on analysis with the Cluster Locator tool (55). Allowance of no intervening genes (max gap = 0) corresponded to 21.64% clustering in the S_GAINED_ gene set, which was 4.27-fold higher than a control group of randomly generated genes (*SI Appendix*, Table S1). Similarly, compared to a random gene set, RW_GAINED_ genes showed 5.58-fold higher clustering when no intervening genes were permitted. Clustering in the S_LOST_ gene set was less pronounced: 1.73-fold over background with a maximum gap tolerance of zero. As expected, an additional control group of random expressed genes in BJ fibroblasts that were unchanged in senescence did not feature any significant gene clustering relative to a default control set that is not subject to a minimum expression threshold. Thus, the up-regulated genes in replicative senescence, in particular, displayed a clustered linear chromosomal distribution that may contribute, in combination with any induced 3D genomic spatial proximity, to their coordinated, enhancer-mediated expression (56, 57).

### NFI, p65, and SMAD2/3 Activate S_GAINED_ Enhancers.

**Fig. 3.**
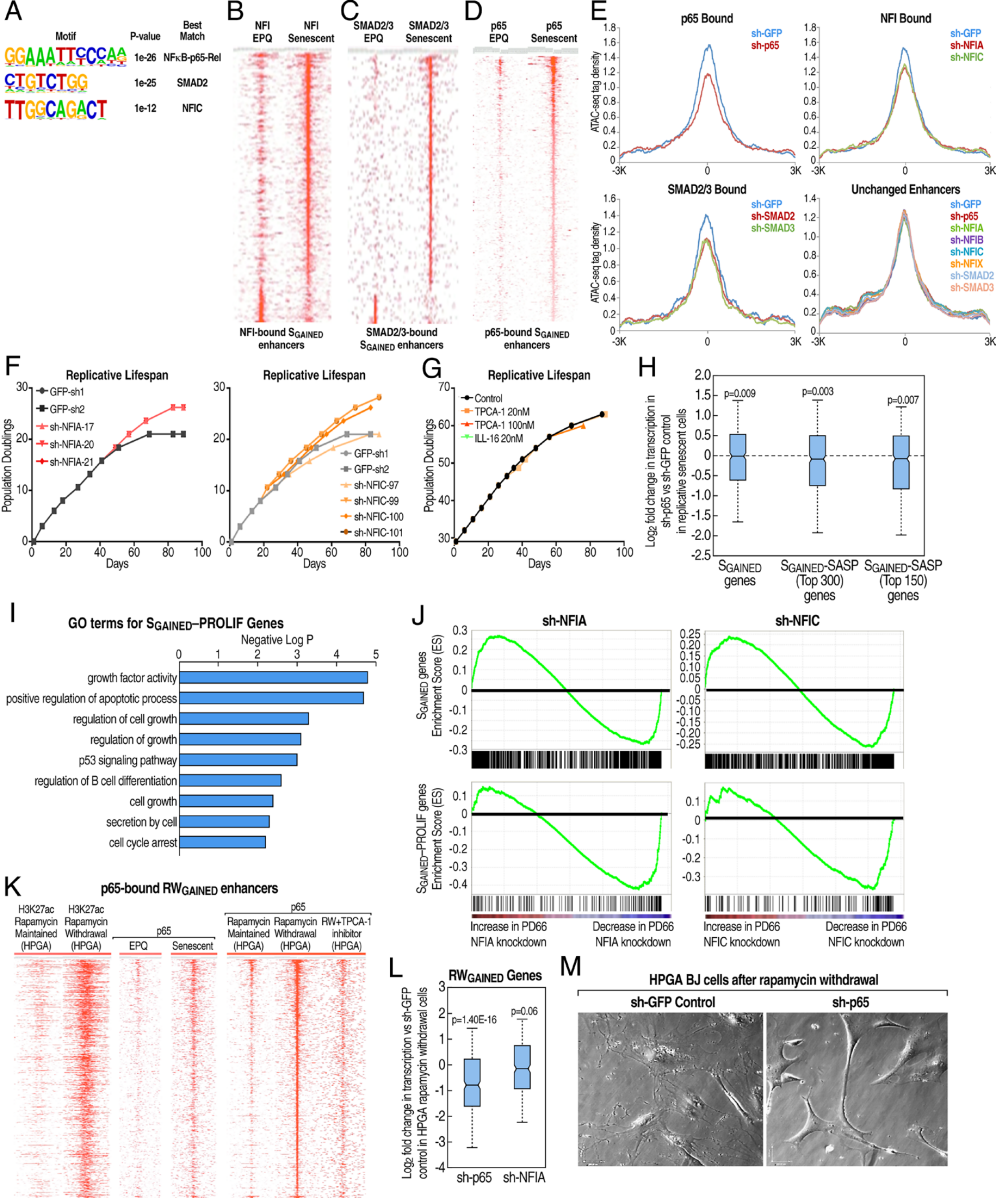
Activation of S_GAINED_ enhancers by NFI, p65, and SMAD2/3. (*A*) HOMER de novo motif analysis of enhancers gained during replicative senescence. (*B*) Heatmap of NFI ChIP-seq enrichment at the NFI-bound subset of S_GAINED_ enhancers in EPQ or senescent BJ fibroblasts. Each column displays a −/+ 3 kb window from the midpoint of the NFI peak. Enhancers were sorted based on the NFI peak score. (*C*) Heatmap of SMAD2/3 ChIP-seq in EPQ or senescent BJ fibroblasts at the SMAD2/3-bound subset of S_GAINED_ enhancers. Each column shows a −/+ 3 kb window from the midpoint of the SMAD2/3 peak. Enhancers were sorted based on the SMAD2/3 peak score. (*D*) Heatmap of p65 ChIP-seq at the p65-bound subset of S_GAINED_ enhancers in EPQ or senescent BJ fibroblasts. Each column represents a −/+ 3 kb window from the midpoint of the p65 peak. Enhancers were sorted based on the p65 peak score. (*E*) Meta-analysis of normalized ATAC-seq tag density, following shRNA-mediated GFP (control) or transcription factor (TF) knockdown, at the indicated TF-bound subset of enhancers featuring gained H3K27ac during replicative senescence. A set of control enhancers with no change in H3K27ac was also analyzed. (*F*) Population doublings over time of BJ fibroblasts after shRNA-mediated stable knockdown of NFI family members versus sh-GFP control. Multiple shRNA constructs targeting indicated NFI family members or GFP were tested in parallel in each assay. (*G*) Population doublings over time of BJ fibroblasts treated continuously with different NF-κB pathway inhibitors or vehicle control. (*H*) Box plot of fold change in RNA-seq tag density for the 639 S_GAINED_ gene set or listed subsets in p65 stable knockdown versus sh-GFP control senescent BJ fibroblasts. Fold change represents the ratio of normalized tag counts in sh-p65 versus sh-GFP control. (*I*) Selected GO-terms from Metascape analysis of the 300-gene S_GAINED_—PROLIF subset. (*J*) GSEA plot of enrichment score for the 639 S_GAINED_ gene set or the 150 S_GAINED_—PROLIF gene subset relative to a background dataset of 14,493 genes exceeding a minimum expression threshold in BJ fibroblasts. Expressed genes on the x-axis were ranked by fold change in knockdown of either NFIA or NFIC versus sh-GFP control in senescent BJ cells. (*K*) Heatmap of p65 or H3K27ac ChIP-seq enrichment at the p65-bound subset of RW_GAINED_ enhancers in indicated conditions. Each column displays a −/+ 3 kb window from the midpoint of the p65 peak. Enhancers were sorted based on the p65 peak score in HPGA rapamycin-withdrawn cells. (*L*) Box plot of change in RNA-seq tag density for RW_GAINED_ genes in p65 or NFIA stable knockdown versus sh-GFP control HPGA BJ fibroblasts after 2 wk of rapamycin withdrawal. Fold change represents the ratio of normalized tag counts in p65 or NFIA knockdown versus sh-GFP control cells. (*M*) Phase contrast images of HPGA BJ fibroblasts with stable shRNA knockdown of p65 or GFP (control) after 2 wk of rapamycin withdrawal.

We next sought to uncover the transcription factors (TFs) that are required for activation of the distinct SASP and proliferation enhancer programs in senescence. De novo motif analysis of S_GAINED_ enhancer peaks revealed significant enrichment of binding sites for three TFs: NF-κB, SMAD2/3, and NFI (Fig. 3*A*). Indeed, NF-κB-mediated inflammation has been extensively linked to aging and senescence (10, 58, 59). SMAD2 and SMAD3 are key TFs in proliferation and other cell activities that interact upon activation by TGFβ family ligands, including Activin A and TGF-β2 (60, 61). Notably, transcripts of *INHBA* and *TGFB2* were up-regulated 2- and 10-fold, respectively, in senescent BJ cells (*SI Appendix*, Fig. S3*A*). The NFI family of TFs, which includes NFIA, NFIB, NFIC, and NIFX, has been reported to impact differentiation, proliferation, and tumorigenesis, and these effects are based, at least in part, on a capacity to alter chromatin accessibility (62, 63).

We performed ChIP-seq to evaluate the distribution of the TFs implicated by motif enrichment. Using a pan-NFI antibody targeting all four NFI family members, we uncovered a marked increase in NFI binding during senescence, from 167 to 418 peaks, at the 2169 S_GAINED_ enhancers (Fig. 3*B* and *SI Appendix*, Fig. S3*B*). ChIP-seq of SMAD2/3 in EPQ and senescent BJ fibroblasts showed a similar increase in binding at S_GAINED_ enhancers, from 69 to 304 peaks (Fig. 3*C* and *SI Appendix*, Fig. S3*C*). ChIP-seq of the NF-κB component p65 also revealed peak accumulation that correlated with population doublings, ultimately resulting in 284 of 2169 S_GAINED_ enhancers exhibiting p65 binding in senescent cells (Fig. 3*D* and *SI Appendix*, Fig. S3 *D* and *E*). This dramatic increase in occupancy of NFI, SMAD2/3, and p65 was conserved in IMR90 cell replicative senescence (*SI Appendix*, Fig. S3 *F*–*H*).

To determine the functional significance of p65, NFI family members, and SMAD2/3 for the transcriptional program regulated by the S_GAINED_ enhancers, we performed Assay for Transposase-Accessible Chromatin using sequencing (ATAC-seq) after 2 wk of shRNA-mediated knockdown of *p65, NFIA, NFIB, NFIC, NFIX, SMAD2,* and *SMAD3* in senescent BJ fibroblasts. While knockdown of *p65, NFIA, NFIC, SMAD2,* and *SMAD3* each resulted in a significant reduction of the ATAC-seq signal at their respective S_GAINED_-bound enhancers, depletion of NFIB or NFIX had comparatively minimal effects (Fig. 3*E* and *SI Appendix*, Fig. S3 *I* and *J*). These ATAC-seq data suggest that binding of p65, NFIA, NFIC, SMAD2, and SMAD3 impacts the activation of the enhancer programs in replicative senescence. Consistently, knockdown of *p65, NFIA*, or *NFIC* reduced the level of transcripts encoded by genes near S_GAINED_ enhancers (*SI Appendix*, Fig. S3*K*). To substantiate the functional contribution of specific NFI family members to a senescent phenotype, we performed selective knockdown of *NFIA, NFIB, NFIC*, or *NFIX* in early-passage BJ fibroblasts, observing that suppression of either *NFIA* or *NFIC* dramatically increased maximum population doublings, whereas a reduction in *NFIB* had no effect and that of *NFIX* actually reduced the maximum number of population doublings (Fig. 3*F* and *SI Appendix*, Fig. S3*L*).

Because withdrawal of rapamycin from high passage BJ fibroblasts elicited a SASP-like profile without restoring proliferation, we reasoned that genes induced in replicative senescence and upon rapamycin withdrawal were likely associated with the SASP. Accordingly, we designated the subset of 150 S_GAINED_ genes that were the most induced following rapamycin withdrawal as S_GAINED_–SASP. Conversely, we predicted that genes up-regulated during replicative senescence but not by rapamycin withdrawal were linked to proliferation control, and we classified the subset of 150 S_GAINED_ genes that were the least induced upon rapamycin withdrawal as S_GAINED_–PROLIF. Whereas pharmacologic inhibition of the p65 pathway had no discernable effect on the maximum population doublings of BJ fibroblasts (Fig. 3*G*), p65 knockdown significantly attenuated S_GAINED_–SASP gene expression (Fig. 3*H*). In contrast, the S_GAINED_–PROLIF genes showed enrichment for GO-terms associated with regulation of growth as well as cell cycle arrest (Fig. 3*I*) and exhibited reduced expression selectively following NFIA or NFIC knockdown in senescent BJ fibroblasts (Fig. 3*J* and *SI Appendix*, Fig. S3 *M* and *N*). Together, these results support the conclusion that NFIA and NFIC promote the antiproliferative, but not the SASP, aspect of replicative senescence.

Next, to elucidate the role of the p65 in the SASP enhancer program of senescence, we performed p65 ChIP-seq in rapamycin-treated HPGA BJ fibroblasts that were either maintained in the presence of the drug or withdrawn for 2 wk. Removal of rapamycin caused a dramatic increase in p65 binding at RW_GAINED_ enhancers, which could be blocked by treatment with the IKKβ inhibitor TCPA-1 (Fig. 3*K*). Knockdown of *p65* also resulted in a striking reduction in RW_GAINED_ gene expression (Fig. 3*L*) and prevented the morphological changes precipitated by rapamycin withdrawal (Fig. 3*M*). In contrast, *NFIA* knockdown did not significantly inhibit RW_GAINED_ gene induction (Fig. 3*L*), further indicating that the proliferative and SASP features of replicative senescence are dependent on separable enhancer activation events.

### Roles of Activin A and TGF-β2 in Regulating the Replicative Senescence Enhancer Programs.

**Fig. 4.**
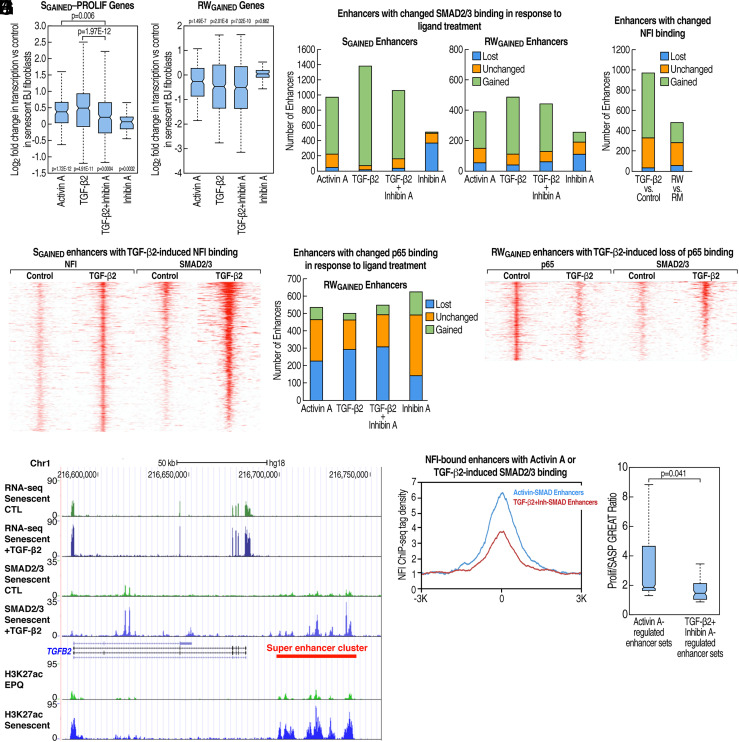
Roles of TGF-β ligands Activin A and TGF-β2 in regulating replicative senescence enhancer programs. (*A* and *B*) Effects of TGF-β ligands on transcription in replicative senescence. Box plots of fold change in RNA-seq tag density for (*A*) 150 S_GAINED_—PROLIF genes or (*B*) 252 RW_GAINED_ genes in replicatively senescent BJ fibroblasts treated for 2 wk with either Activin A, TGF-β2, TGF-β2+Inhibin A, or Inhibin A versus DMSO control. (*C*) Stacked bar graph quantifying S_GAINED_ or RW_GAINED_ enhancers with no change or at least a twofold increase or decrease in SMAD2/3 binding in senescent or HPGA rapamycin-withdrawn BJ fibroblasts, respectively, treated with Activin A, TGF-β2, TGF-β2+Inhibin A, or Inhibin A versus DMSO control. (*D*) Stacked bar graph quantifying S_GAINED_ enhancers in senescent BJ fibroblasts treated with TGF-β2 versus DMSO control (*Left*) or RW_GAINED_ enhancers in rapamycin-withdrawn versus rapamycin-maintained HPGA BJ fibroblasts (*Right*) with no change or at least a twofold increase or decrease in NFI binding. (*E*) Heatmap of normalized NFI and SMAD2/3 ChIP-seq tag density in replicatively senescent BJ fibroblasts after 2-wk treatment with vehicle control or TGF-β2. Plots were centered on the subset of S_GAINED_ enhancers showing >twofold increase in NFI tag density upon TGF-β2 treatment and sorted by fold change in SMAD2/3 binding in response to TGF-β2 versus vehicle control (normalized to outer regions of −/+ 3 kb window). (*F*) Stacked bar graph quantifying RW_GAINED_ enhancers with no change or at least a twofold increase or decrease in p65 binding in rapamycin-withdrawn HPGA BJ fibroblasts treated with Activin A, TGF-β2, TGF-β2+Inhibin A, or Inhibin A versus DMSO control. (*G*) Heatmap of normalized p65 and SMAD2/3 ChIP-seq tag density in HPGA BJ fibroblasts treated with vehicle control or TGF-β2 during 2 wk-withdrawal from rapamycin. Plots were centered on the subset of RW_GAINED_ enhancers showing >twofold decrease in p65 tag density upon TGF-β2 treatment and sorted by fold change in SMAD2/3 binding in response to TGF-β2 versus vehicle control (normalized to outer regions of −/+ 3 kb window). (*H*) UCSC genome browser tracks of ChIP-seq and RNA-seq profiles at the *TGFB2* locus. (*I*) Meta-analysis of normalized NFI ChIP-seq tag density at Activin A or TGF-β2+Inhibin A treatment-specific SMAD2/3-bound enhancers in replicatively senescent BJ fibroblasts (normalized to DMSO control distribution). Plots were centered on enhancers with >twofold increase in SMAD2/3 ChIP-seq tag density in both senescent and rapamycin-withdrawn HPGA BJ fibroblasts. (*J*) Box plot of the Prolif/SASP GREAT ratios of 11 paired Activin A- and TGF-β2+Inhibin A-regulated enhancer sets (see *SI Appendix,* Table S2 for list of enhancer sets and *SI Appendix*, *Extended Methods* for description). *P*-value was calculated using a Wilcoxon signed rank test.

Treatment with TGFβ family ligands revealed that activation of SMAD2/3 transcriptionally regulates the S_GAINED_–PROLIF gene cohort (Fig. 4*A*) as well as the p65-dependent S_GAINED_–SASP and RW_GAINED_ gene programs (Fig. 4*B* and *SI Appendix*, Fig. S4*A*). TGF-β2 and Activin A stimulation increased SMAD2/3 binding at both S_GAINED_ and RW_GAINED_ enhancers (Fig. 4*C*). This increased SMAD2/3 presence at S_GAINED_ enhancers coincides with a dramatic induction of NFI co-occupancy (Fig. 4 *D* and *E*). In contrast, SMAD2/3 binding at RW_GAINED_ enhancers prevents p65 co-occupancy (Fig. 4 *F* and *G*). These data indicate that SMAD2/3 transcriptionally regulate the two enhancer programs of senescence by promoting NFI cobinding to block proliferation and by limiting p65 activity to suppress the SASP.

TGF-β2 and Activin A initiate a signaling cascade by binding type II receptors, which activate signal-propagating type I receptors via phosphorylation, ultimately leading to activation of the SMAD2/3 complex (60). TGF-β2 binds exclusively to the type I receptor TGFBR1 and the type II receptor TGFBR2, whereas a homodimer of *INHBA-*encoded Activin A binds the type II receptors ActRII and ActRIIB with high affinity and preferentially interacts with the type I receptor ActRIB (also known as ALK4) (60, 61). Alternatively, the *INHBA* protein product can heterodimerize with the *INHA*-encoded protein to produce Inhibin A, which competitively inhibits the activity of Activin A by multiple mechanisms involving binding or sequestration of its type I and II receptor(s), respectively, thereby preventing downstream phosphorylation-associated activation of SMAD2/3 (61).

Upon scrutiny of the *TGFB2* and *INHBA* transcription units, we noted the presence of proximal super-enhancers that displayed elevated H3K27ac signal and SMAD2/3 binding in senescent cells (Fig. 4*H* and *SI Appendix*, Fig. S4*B*). Treatment with either Activin A or TGF-β2 resulted in a twofold to threefold increase of *TGFB2* and *INHBA* transcripts (*SI Appendix*, Fig. S4*C*), suggesting reciprocal feedback regulation. To assess the generality of this regulatory scheme, we analyzed publicly available RNA-seq datasets derived from a variety of cell types treated with Activin A, TGF-β1, or TGF-β2, which suggested conservation of the feedback loops controlling *INHBA* and *TGFB2* expression (*SI Appendix*, Fig. S4*D*). This analysis also revealed that TGF-β1 stimulation can strongly induce *INHBA* and *TGFB2* under most conditions; however, there was no indication of *TGFB1* autoregulation.

To circumvent this positive feedback loop connecting TGF-β2 and Activin A, we exploited the selective suppressive capacity of Inhibin A toward the latter (61). Cotreatment of senescent BJ cells with Inhibin A and TGF-β2 resulted in significantly decreased activation of S_GAINED_—PROLIF genes relative to either Activin A or TGF-β2 alone (Fig. 4*A*). However, compared to treatment with TGF-β2 by itself, TGF-β2+Inhibin A did not differentially affect S_GAINED_—SASP or RW_GAINED_ genes (Fig. 4*B* and *SI Appendix*, Fig. S4*A*). These results suggest that Activin A selectively activates a SMAD2/3 program supporting the proliferative arrest phenotype of senescence, while TGF-β2 induces an alternative SMAD2/3 program that functions to suppress the SASP. To explore the molecular mechanism underlying this phenomenon, we performed SMAD2/3 ChIP-seq on senescent cells treated with TGF-β2 only or cotreated with Inhibin A and TGF-β2, which revealed a decrease in SMAD2/3 peaks at S_GAINED_ enhancers in the latter scenario (Fig. 4*C*). In addition, compared to TGF-β2 alone, cotreatment with Inhibin A and TGF-β2 had minimal impact on SMAD2/3 binding (Fig. 4*C*) and no effect on p65 occupancy at RW_GAINED_ enhancers (Fig. 4*F*). These findings are consistent with our earlier observation that blocking Activin A signaling does not appreciably affect SMAD2/3-mediated suppression of the SASP program. Furthermore, for SMAD2/3-bound enhancers in senescent cells, those whose recruitment of SMAD2/3 was selectively induced by Activin A, as opposed to TGF-β2+Inhibin A, exhibited dramatically higher NFI co-occupancy (Fig. 4*I* and *SI Appendix*, Fig. S4*E*).

To substantiate the functional distinction of the differentially regulated, SMAD2/3-bound enhancer cohorts, we performed GREAT analysis on multiple sets of Activin A- or TGF-β2+Inhibin A-responsive enhancers (*SI Appendix*, *Extended Methods*). We observed a statistically significant association of Activin A-regulated enhancers with GO terms related to proliferation and cell cycle whereas TGF-β2+Inhibin A-regulated enhancers showed enrichment in GO categories pertaining to secretion, cytokines, and inflammation (Fig. 4*J* and *SI Appendix*, Fig. S4*F*). Additionally, using 83 enhancer sets associated with either the Activin A/proliferation arrest or TGF-β2+InhibinA/SASP pathways, we tested for enrichment of aging pathology GWAS/PheWAS trait SNPs (39, 40). Again, we observed clear clustering of TGF-β2+Inhibin A/SASP-related enhancer sets with traits linked to chronic inflammation, including C-reactive protein levels, acute myeloid leukemia, and brain aneurysm (*SI Appendix*, Fig. S4*G*). In contrast, Activin A/proliferation arrest-associated enhancer sets were enriched for SNPs connected to body mass index (*SI Appendix*, Fig. S4*G*). Collectively, our results provide compelling evidence for two functionally distinct SMAD2/3-signaling pathways in replicative senescence triggered by different TGF-β family ligands: one involving Activin A induction of NFI enhancer binding to restrict proliferation and the other featuring reduced p65 enhancer occupancy elicited by TGF-β2 that suppresses the SASP.

## Discussion

**Fig. 5.**
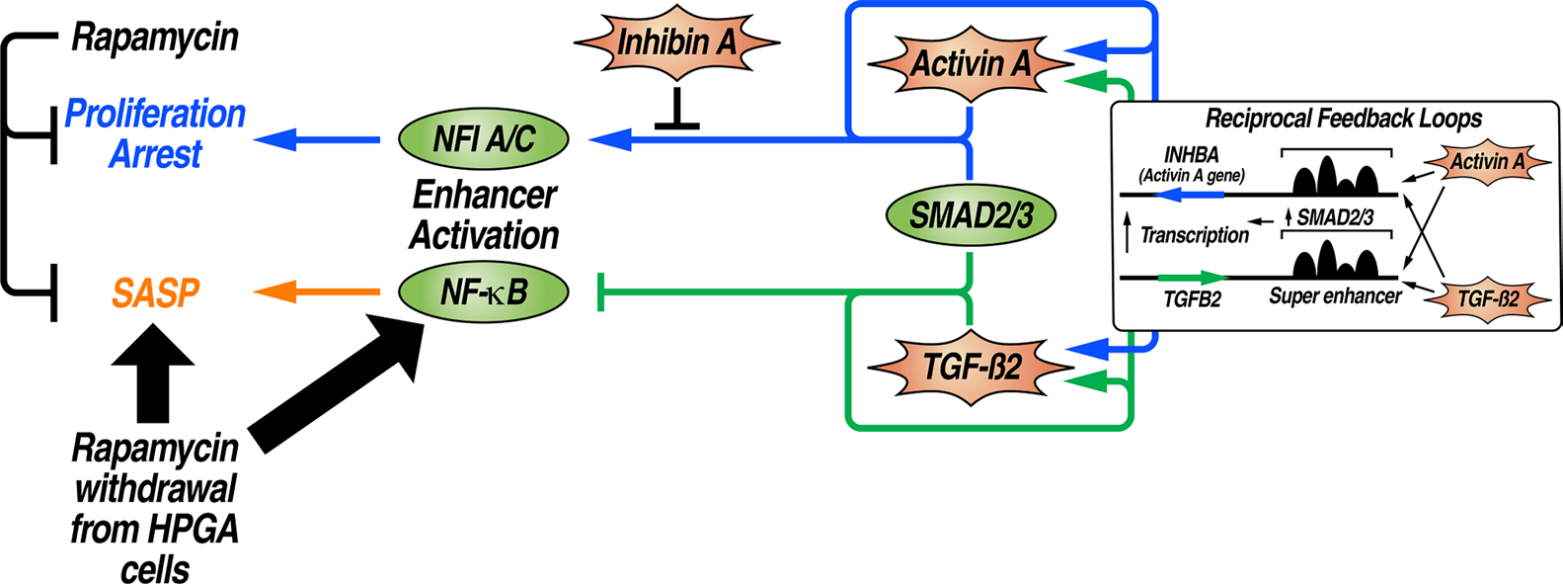
Model of separable enhancer programs in replicative senescence that are differentially responsive to TGF-β family ligands and rapamycin withdrawal. Activin A stimulates SMAD2/3 collaboration with NFIA/C at enhancers controlling proliferation arrest, while TGF-β2 activates SMAD2/3 to block requisite NF-κB binding of enhancers that drive the SASP. Both senescent enhancer programs can be subdued by rapamycin treatment; however, rapamycin withdrawal causes robust activation of the NF-κB-dependent SASP enhancer cohort and associated proinflammatory gene transcription. Notably, the transcription units for Activin A (*INHBA*) and TGF-β2 are under super-enhancer control and are subject to reciprocal feedback regulation.

Our findings support the hypothesis that an altered enhancer activation profile serves as the underlying driver of characteristic phenotypes in replicative senescence, permitting the identification of key ligands as well as novel TFs that specifically and differentially impact the SASP and proliferative transcriptional programs. By leveraging data regarding the effects of rapamycin and its withdrawal, we have uncovered two separate cohorts of enhancers that dictate the features of replicative senescence: One is activated by NFIA and NFIC to halt proliferation while the other involves p65-mediated SASP induction. These enhancer subsets and their activating TFs are controlled by SMAD2/3 in a ligand-specific manner, with Activin A preferentially inducing NFI binding to establish proliferative repression and TGF-β2 committed to inhibiting p65 occupancy to attenuate the SASP. We have also described an unappreciated feedback loop that links the expression of TGF-β2 and Activin A, which may have previously obscured their functional distinction in cellular senescence (Fig. 5).

The capacity of rapamycin to delay cellular senescence and extend model organism lifespan across phyla is supported by the preponderance of evidence, accumulated over more than a decade (47–53), and multiple clinical trials are currently underway to test its therapeutic application in various aging-related disorders (64). Moreover, there are now many reports of health span and longevity enhancements from intermittent as well as transient rapamycin treatment of aged animals, which may also largely eliminate adverse side effects (65, 66). Our findings regarding rapamycin withdrawal from growth-arrested cells reveal an unexpected feature of long-term rapamycin treatment at the cellular level. While rapamycin suppresses changes in enhancer activation and gene expression that define high-passage senescent fibroblasts, its withdrawal from growth-arrested cells following extended administration causes a rapid and dramatic augmentation of the SASP, mediated by robust activation of a conserved enhancer cohort and the linked transcriptional program. The molecular mechanism(s) and cell biology underlying this phenomenon remain to be determined, including the potential involvement of various cellular processes regulated by mTOR signaling and/or the DNA damage response triggered by shortened telomeres.

A definitive role for NFI proteins in senescence has not been previously established. While our study demonstrated a clear association of NFIA and NFIC with replicative senescence, the basis for the apparent differential involvement of NFI family members, which display a high level of conservation in their DNA-binding domains (67), is unclear. Furthermore, as NFIA and NFIC are seemingly nonredundant in replicative senescence, whether they collaborate, for example via the formation of functional heterodimers (68), or, alternatively, are utilized in a distinct fashion at proliferation-associated enhancers will require further dissection. It also remains to be determined whether the same NFI proteins selectively impact other types of senescence. NFI TFs have previously been linked to development (63), with haploinsufficiency of NFIA, NFIB, and NFIX resulting in overgrowth phenotypes (69), as well as oncogenesis (62). Our findings here, along with recent reports (62, 70), suggest that particular NFI family members may also serve as key mediators of aging-associated human pathology. Multiple TFs have previously been implicated in gene activation events in aging as well as cellular senescence (43, 44, 59, 71), and, in a few instances, such as for C/EBPα (44), p65, and AP-1 (43), crucial roles in driving senescent enhancer-mediated gene regulation have been documented. AP-1 has been reported to serve as a principal pioneer factor at senescent enhancers to regulate SASP-related gene expression in particular. Accordingly, AP-1 facilitates the enhancer occupancy of p65 and other TFs by increasing the chromatin accessibility at their cognate DNA-binding motifs (43). Thus, the significance of the pioneering function of NFIA/C specifically at proliferation-associated senescent enhancers warrants additional investigation.

TGF-β signaling has been extensively studied in the context of aging-related pathology and cellular senescence, as TGF-β family ligands are well-known SASP constituents (72). Notably, individual shRNA-mediated knockdown of the ALK4, ALK5, or ALK7 receptor, which are engaged by TGF-β or activin isoforms in a quasi-selective manner (73), partially rescued paracrine senescence induced by conditioned medium from RAS-expressing senescent IMR90 fibroblasts. These findings clearly implicated canonical TGF-β signaling involving SMAD2/3 phospho-activation in the establishment and dissemination of cellular senescence (74). However, unique roles for particular TGF-β ligands in modulating enhancer activity via SMAD2/3 binding have not been elucidated in senescent cells. Indeed, an enhancer-dependent feedback loop between Activin A and TGF-β2 may have masked the ligand-specific activity of SMAD2/3. We show that treatment with either ligand results in activation of super-enhancer clusters that regulate the genes encoding each of the ligands. Yet, the Activin A-associated effects are phenotypically, and potentially pathologically, distinct from those of TGF-β2, and the indirect TGF-β2-mediated induction of Activin A signaling can be specifically overcome by combining TGF-β2 with Inhibin A.

Given the context-dependent and pleotropic effects of Activin A and TGF-β in age-related disease, including cancer (73, 75–83), systemic administration of TGF-β ligands might differentially affect aging tissues. Moreover, most ongoing clinical trials related to TGF-β signaling involve interventions designed to suppress its activation. Nevertheless, a well-tolerated dietary supplement containing TGF-β2 is being tested in adults with Crohn’s disease (CD) after showing efficacy in treating pediatric CD (84). In addition, a ligand trap targeting Activin A demonstrated clinical benefit for cancer cachexia (85). While our finding of distinct senescence-related effects of Activin A and TGF-β2 suggests that selectively activating the signaling program specific to the latter while limiting that of the former could have clinical utility, especially in tissues impacted by cellular senescence that can be specifically targeted, the therapeutic potential of combined TGF-β2 and Inhibin A treatment is unclear at present in the absence of rigorous in vivo validation.

Mechanistically, the precise biochemical signal transduction events that underlie the unique SMAD2/3-binding programs activated by TGF-β2 and Activin A remain unsolved. Because Activin A and TGF-β signaling can induce a variety of posttranslational modifications of the SMAD2/3 complex, including phosphorylation, poly- and monoubiquitination, acetylation, and poly(ADP-ribosyl)ation (86), the possibility of distinct TGF-β ligand-elicited covalent marks on SMAD2/3 in senescent cells deserves future exploration. Differentially modified SMAD2/3 complexes that selectively associate with other TFs could stabilize *cis* interactions or facilitate *trans* binding at certain enhancers and thus may represent innovative therapeutic targets. Detailed mechanistic studies might also help to explain why TGF-β2-induced SMAD2/3 enhancer binding is unconducive to p65 co-occupancy, and, more broadly, how enhancer–promoter specificity is achieved in replicative senescence (87). Overall, the data presented here link specific TFs to signaling pathways that regulate distinct enhancer cohorts dictating the characteristic features of replicative senescence with potentially actionable implications for normal and pathological aging.

## Materials and Methods

Extended methods pertaining to tissue culture, shRNA constructs, virus production and transduction conditions, SA-β-gal staining, microscopy, RT-qPCR, ChIP-seq, GRO-seq, ATAC-seq, deep-seq data processing, determination of gene expression (sub)sets, in-situ Hi-C procedure/data analysis, gene clustering assessment, utilization of publicly available RNA-seq datasets, GREAT meta-analysis, and GWAS/PheWAS analysis are provided in *SI Appendix*.

### Tissue Culture.

Human BJ (ATCC® CRL-2522) foreskin and IMR90 (ATCC CCL-186) lung fibroblasts were cultured at 37 °C in 20% O_2_ and 5% CO_2_ using high-glucose DMEM (4.5 g/L, Gibco 10566-016) supplemented with 10% FBS. Cell lines were initially plated at PD26, grown to confluence, and then split 1:8. Population doubling was recorded and incremented by 3 at each 1:8 passage.

BJ fibroblasts were deemed “early passage” prior to population doubling (PD) 35 after initial thawing. Intermediate fibroblasts were harvested between PD35 and 50. Fibroblasts were considered senescent when they failed to divide within 1 wk of passaging, which typically occurred after at least PD64 and was initially confirmed using SA-β-gal staining.

To mitigate the effects of cell cycle, nonsenescent, dividing fibroblasts were grown to confluence prior to harvesting for any experiments, resulting in contact-inhibited quiescence and similar growth arrest among all populations of cells. Early passage cells collected in this manner are referred to as early passage quiescent (EPQ).

For rapamycin treatments, cells were maintained in 10 nM rapamycin (Tocris-1292) after recovery from thawing. Rapamycin-maintained cells were collected for RNA-seq and ChIP-seq at PD31 and PD66 to correspond with similar PD for EPQ and senescent non-rapamycin-treated cells. For rapamycin withdrawal experiments, rapamycin-maintained cells were grown to proliferation arrest, which typically occurred between PD75 and 85. These cells were referred to as High Passage Growth Arrested (HPGA). At this point, the cells were divided into two plates, with rapamycin treatment maintained in one and withdrawn from the other until harvesting 2 wk later.

For short-term senescence experiments, senescent (>PD60) BJ fibroblasts were treated with SMAD2/3-regulating ligands Activin A (R&D Systems—338-AC-010), Inhibin A (R&D Systems—8506-AB-010), TGF-β2 (R&D Systems—302-B2-010), or combined TGF-β2+Inhibin A for 2 wk, after which the cells were harvested. For short-term rapamycin withdrawal experiments, HPGA BJ fibroblasts maintained in 10 nM rapamycin were treated with the above ligands or IKK inhibitor TPCA-1 (500 nM) for 14 d, after which the cells were split into 2 wells, maintained in the indicated drug/ligand treatments, and either maintained in or withdrawn from 10 nM rapamycin treatment.

### Enhancer-Targeting shRNA Vectors.

Stable knockdown of enhancer RNAs (eRNAs) was performed with shRNAs in the pLKO.1 expression vector. For enhancer knockdowns, we selected eRNAs that were highly induced during replicative senescence (based on GRO-seq) at enhancers that were strongly activated in this context (based on H3K4me^2^ and H3K27ac tag density). We also stipulated that targeted enhancers were located within 100 kb of a gene that was robustly transcriptionally up-regulated during replicative senescence. From these eRNA-generating enhancer regions, specific target sequences were determined using either Dharmacon Custom siRNA or DSIR (88) web applications.

### Antibodies.

The following antibodies were used in this publication: SMAD2/3, Santa Cruz sc-6032 (N19) and sc-133098 (C-8) in an equimolar cocktail; NFI, Santa Cruz sc-5567 (H-300); p65, Santa Cruz sc-372 (C-20); H3K4me^2^, Abcam ab7766; H3K27ac, Abcam ab4729.

### RNA-seq.

Total cellular RNA was harvested from cells using the RNeasy Mini Kit (QIAGEN-74104), including the DNase treatment step. Purified RNA was prepared for sequencing with the TruSeq Stranded Total RNA LT Sample Prep Kit (RS-122-2201/RS-122-2202) for ribosomal RNA-depleted libraries. Samples were run on the Illumina HiSeq 4000 as single-read reactions of 50 or 75 cycles.

### ChIP-seq.

Chromatin immunoprecipitation assays were performed as previously described (89) with some modifications. See *SI Appendix*, *Extended Methods* for a detailed description.

Purified chromatin from histone and transcription factor ChIPs was prepared for deep sequencing with the KAPA LTP Library Preparation Kit (KK8232) and the KAPA Hyper Prep Kit (KK8500), respectively, following the manufacturer’s instructions. Samples were run on the Illumina HiSeq 4000 as single-read reactions of 50 or 75 cycles.

### Processing of Deep Sequencing Data.

Aligned deep-seq reads were processed and analyzed using various programs in the HOMER software package (90), as detailed in *SI Appendix*, *Extended Methods*.

### Box Plot Information.

Box plots indicate the population median value (indented center line), 25th and 75th percentiles (bottom and top edges of boxes, respectively), and 5th and 95th percentiles (ends of lower and upper whiskers, respectively).

### Determination of Enhancer Sets.

Altered enhancers in either replicative senescence (S_GAINED_ and S_LOST_) or rapamycin withdrawal (RW_GAINED_) were those that displayed >twofold change in H3K27ac and >1.5-fold change in H3K4me^2^ tag counts between the conditions being considered. Unless noted otherwise, altered enhancers were required to reach these thresholds in each of two biological replicates for both histone marks.

### Significance Testing of Enhancer and Gene Populations.

To determine whether different sets of fold change distributions (typically presented in box plot figures), including those for RNA-seq, GRO-seq, ATAC-seq, and ChIP-seq, are significantly different from either each other or from 0 (i.e., that the distributions show a significant directional change), two-tailed z-tests were performed using the median-normalized log_2_ fold change in tag density across enhancer or transcript coordinates (*SI Appendix*, *Extended Methods*). The output *P*-values are reported in each graph next to the relevant comparisons. Population sizes were defined by the gene or enhancer (sub)set analyzed in each panel.

## Supplementary Material

Appendix 01 (PDF)

## Data Availability

The functional genomics sequencing data for this paper have been deposited as Gene Expression Omnibus (GEO) Series GSE146585 and are accessible at https://www.ncbi.nlm.nih.gov/geo/query/acc.cgi?acc=GSE146585 (46).

## References

[1] M. P. Baar , Targeted apoptosis of senescent cells restores tissue homeostasis in response to chemotoxicity and aging. Cell **169**, 132–147.e16 (2017). 28340339 10.1016/j.cell.2017.02.031 PMC5556182

[2] D. J. Baker , Naturally occurring p16Ink4a-positive cells shorten healthy lifespan. Nature **530**, 184–189 (2016). 26840489 10.1038/nature16932 PMC4845101

[3] J. Chang , Clearance of senescent cells by ABT263 rejuvenates aged hematopoietic stem cells in mice. Nat. Med. **22**, 78–83 (2016). 26657143 10.1038/nm.4010 PMC4762215

[4] V. Gorgoulis , Cellular senescence: Defining a path forward. Cell **179**, 813–827 (2019). 31675495 10.1016/j.cell.2019.10.005

[5] P. D. Adams, Healing and hurting: Molecular mechanisms, functions, and pathologies of cellular senescence. Mol. Cell **36**, 2–14 (2009). 19818705 10.1016/j.molcel.2009.09.021

[6] J.-P. Coppé, P.-Y. Desprez, A. Krtolica, J. Campisi, The senescence-associated secretory phenotype: The dark side of tumor suppression. Annu. Rev. Pathol. **5**, 99–118 (2010). 20078217 10.1146/annurev-pathol-121808-102144 PMC4166495

[7] D. Muñoz-Espín, M. Serrano, Cellular senescence: From physiology to pathology. Nat. Rev. Mol. Cell Biol. **15**, 482–496 (2014). 24954210 10.1038/nrm3823

[8] J. Campisi, F. d’Adda di Fagagna, Cellular senescence: When bad things happen to good cells. Nat. Rev. Mol. Cell Biol. **8**, 729–740 (2007). 17667954 10.1038/nrm2233

[9] D. J. Baker , Clearance of p16Ink4a-positive senescent cells delays ageing-associated disorders. Nature **479**, 232–236 (2011). 22048312 10.1038/nature10600 PMC3468323

[10] A. Freund, C. K. Patil, J. Campisi, p38MAPK is a novel DNA damage response-independent regulator of the senescence-associated secretory phenotype. EMBO J. **30**, 1536–1548 (2011). 21399611 10.1038/emboj.2011.69 PMC3102277

[11] D. V. Faget, Q. Ren, S. A. Stewart, Unmasking senescence: Context-dependent effects of SASP in cancer. Nat. Rev. Cancer **19**, 439–453 (2019). 31235879 10.1038/s41568-019-0156-2

[12] J. M. van Deursen, The role of senescent cells in ageing. Nature **509**, 439–446 (2014). 24848057 10.1038/nature13193 PMC4214092

[13] J. C. Jeyapalan, J. M. Sedivy, Cellular senescence and organismal aging. Mech. Ageing Dev. **129**, 467–474 (2008). 18502472 10.1016/j.mad.2008.04.001 PMC3297662

[14] C. López-Otín, M. A. Blasco, L. Partridge, M. Serrano, G. Kroemer, Hallmarks of aging: An expanding universe. Cell **186**, 243–278 (2023). 36599349 10.1016/j.cell.2022.11.001

[15] J. Campisi, J. K. Andersen, P. Kapahi, S. Melov, Cellular senescence: A link between cancer and age-related degenerative disease? Semin. Cancer Biol. **21**, 354–359 (2011). 21925603 10.1016/j.semcancer.2011.09.001 PMC3230665

[16] B. G. Childs , Senescent intimal foam cells are deleterious at all stages of atherosclerosis. Science **354**, 472–477 (2016). 27789842 10.1126/science.aaf6659 PMC5112585

[17] O. H. Jeon , Local clearance of senescent cells attenuates the development of post-traumatic osteoarthritis and creates a pro-regenerative environment. Nat. Med. **23**, 775–781 (2017). 28436958 10.1038/nm.4324 PMC5785239

[18] M. Xu , Senolytics improve physical function and increase lifespan in old age. Nat. Med. **24**, 1246–1256 (2018). 29988130 10.1038/s41591-018-0092-9 PMC6082705

[19] G. Casella , Transcriptome signature of cellular senescence. Nucleic Acids Res. **47**, 7294–7305 (2019). 31251810 10.1093/nar/gkz555 PMC6698740

[20] A. D. Hudgins , Age- and tissue-specific expression of senescence biomarkers in mice. Front. Genet. **9**, 59 (2018). 29527222 10.3389/fgene.2018.00059 PMC5829053

[21] L. N. Booth, A. Brunet, The aging epigenome. Mol. Cell **62**, 728–744 (2016). 27259204 10.1016/j.molcel.2016.05.013 PMC4917370

[22] T. Chandra , Global reorganization of the nuclear landscape in senescent cells. Cell Rep. **10**, 471–483 (2015). 25640177 10.1016/j.celrep.2014.12.055 PMC4542308

[23] S. W. Criscione , Reorganization of chromosome architecture in replicative cellular senescence. Sci. Adv. **2**, e1500882 (2016). 26989773 10.1126/sciadv.1500882 PMC4788486

[24] R. P. McCord , Correlated alterations in genome organization, histone methylation, and DNA-lamin A/C interactions in Hutchinson-Gilford progeria syndrome. Genome Res. **23**, 260–269 (2013). 23152449 10.1101/gr.138032.112 PMC3561867

[25] H. A. Cruickshanks , Senescent cells harbor features of the cancer epigenome. Nat. Cell Biol. **15**, 1495–1506 (2013). 24270890 10.1038/ncb2879 PMC4106249

[26] S. A. Evans, J. Horrell, N. Neretti, The three-dimensional organization of the genome in cellular senescence and age-associated diseases. Semin. Cell Dev. Biol. **90**, 154–160 (2019). 30031215 10.1016/j.semcdb.2018.07.022 PMC6661233

[27] P. P. Shah , Lamin B1 depletion in senescent cells triggers large-scale changes in gene expression and the chromatin landscape. Genes Dev. **27**, 1787–1799 (2013). 23934658 10.1101/gad.223834.113 PMC3759695

[28] M. De Cecco , L1 drives IFN in senescent cells and promotes age-associated inflammation. Nature **566**, 73–78 (2019). 30728521 10.1038/s41586-018-0784-9 PMC6519963

[29] L. Dupays, T. Mohun, Spatiotemporal regulation of enhancers during cardiogenesis. Cell. Mol. Life Sci. **74**, 257–265 (2017). 27497925 10.1007/s00018-016-2322-y PMC5219004

[30] S. Heinz, C. E. Romanoski, C. Benner, C. K. Glass, The selection and function of cell type-specific enhancers. Nat. Rev. Mol. Cell Biol. **16**, 144–154 (2015). 25650801 10.1038/nrm3949 PMC4517609

[31] D. Hnisz , Convergence of developmental and oncogenic signaling pathways at transcriptional super-enhancers. Mol. Cell **58**, 362–370 (2015). 25801169 10.1016/j.molcel.2015.02.014 PMC4402134

[32] H. S. Kim , Pluripotency factors functionally premark cell-type-restricted enhancers in ES cells. Nature **556**, 510–514 (2018). 29670286 10.1038/s41586-018-0048-8 PMC6021123

[33] H. K. Long, S. L. Prescott, J. Wysocka, Ever-changing landscapes: Transcriptional enhancers in development and evolution. Cell **167**, 1170–1187 (2016). 27863239 10.1016/j.cell.2016.09.018 PMC5123704

[34] E. Smith, A. Shilatifard, Enhancer biology and enhanceropathies. Nat. Struct. Mol. Biol. **21**, 210–219 (2014). 24599251 10.1038/nsmb.2784

[35] J. D. Brown , NF-kB directs dynamic super enhancer formation in inflammation and atherogenesis. Mol. Cell **56**, 219–231 (2014). 25263595 10.1016/j.molcel.2014.08.024 PMC4224636

[36] J. D. Brown , BET bromodomain proteins regulate enhancer function during adipogenesis. Proc. Natl. Acad. Sci. U.S.A. **115**, 2144–2149 (2018). 29444854 10.1073/pnas.1711155115 PMC5834672

[37] I. Hazan, J. Monin, B. A. M. Bouwman, N. Crosetto, R. I. Aqeilan, Activation of oncogenic super-enhancers is coupled with DNA repair by RAD51. Cell Rep. **29**, 560–572.e4 (2019). 31618627 10.1016/j.celrep.2019.09.001 PMC6899447

[38] B. R. Tennant , The TrxG complex mediates cytokine induced de novo enhancer formation in islets. PLoS One **10**, e0141470 (2015). 26505193 10.1371/journal.pone.0141470 PMC4623983

[39] S. C. Johnson, X. Dong, J. Vijg, Y. Suh, Genetic evidence for common pathways in human age-related diseases. Aging Cell **14**, 809–817 (2015). 26077337 10.1111/acel.12362 PMC4568968

[40] S. C. Johnson , Network analysis of mitonuclear GWAS reveals functional networks and tissue expression profiles of disease-associated genes. Hum. Genet. **136**, 55–65 (2017). 27704213 10.1007/s00439-016-1736-9 PMC5214989

[41] B. A. Benayoun , Remodeling of epigenome and transcriptome landscapes with aging in mice reveals widespread induction of inflammatory responses. Genome Res. **29**, 697–709 (2019). 30858345 10.1101/gr.240093.118 PMC6442391

[42] N. Tasdemir , BRD4 connects enhancer remodeling to senescence immune surveillance. Cancer Discov. **6**, 612–629 (2016). 27099234 10.1158/2159-8290.CD-16-0217 PMC4893996

[43] R. I. Martínez-Zamudio , AP-1 imprints a reversible transcriptional programme of senescent cells. Nat. Cell Biol. **22**, 842–855 (2020). 32514071 10.1038/s41556-020-0529-5 PMC7899185

[44] Y. Guan , Senescence-activated enhancer landscape orchestrates the senescence-associated secretory phenotype in murine fibroblasts. Nucleic Acids Res. **48**, 10909–10923 (2020). 33045748 10.1093/nar/gkaa858 PMC7641768

[45] A. Subramanian , Gene set enrichment analysis: A knowledge-based approach for interpreting genome-wide expression profiles. Proc. Natl. Acad. Sci. U.S.A. **102**, 15545–15550 (2005). 16199517 10.1073/pnas.0506580102 PMC1239896

[46] T. Suter, M. G. Rosenfeld, “Functional genomics assays, including ChIP-seq, ATAC-seq, RNA-seq, GRO-seq, and *in situ* Hi-C, in the context of replicative senescence”. GEO. https://www.ncbi.nlm.nih.gov/geo/query/acc.cgi?acc=GSE146585. Deposited 6 March 2020.

[47] S. C. Johnson, P. S. Rabinovitch, M. Kaeberlein, mTOR is a key modulator of ageing and age-related disease. Nature **493**, 338–345 (2013). 23325216 10.1038/nature11861 PMC3687363

[48] D. E. Harrison , Rapamycin fed late in life extends lifespan in genetically heterogeneous mice. Nature **460**, 392–395 (2009). 19587680 10.1038/nature08221 PMC2786175

[49] V. D. Longo, Programmed longevity, youthspan, and juventology. Aging Cell **18**, e12843 (2019). 30334314 10.1111/acel.12843 PMC6351819

[50] R. A. Miller , Rapamycin, but not resveratrol or simvastatin, extends life span of genetically heterogeneous mice. J. Gerontol. A. Biol. Sci. Med. Sci. **66**, 191–201 (2011). 20974732 10.1093/gerona/glq178 PMC3021372

[51] J. E. Wilkinson , Rapamycin slows aging in mice. Aging Cell **11**, 675–682 (2012). 22587563 10.1111/j.1474-9726.2012.00832.x PMC3434687

[52] C. Lerner , Reduced mammalian target of rapamycin activity facilitates mitochondrial retrograde signaling and increases life span in normal human fibroblasts. Aging Cell **12**, 966–977 (2013). 23795962 10.1111/acel.12122 PMC5559196

[53] T. V. Pospelova , Rapamycin induces pluripotent genes associated with avoidance of replicative senescence. Cell Cycle **12**, 3841–3851 (2013). 24296616 10.4161/cc.27396 PMC3905076

[54] A. Zirkel , Hmgb2 loss upon senescence entry disrupts genomic organization and induces CTCF clustering across cell types. Mol. Cell **70**, 730–744.e6 (2018). 29706538 10.1016/j.molcel.2018.03.030

[55] F. Pazos Obregón , Cluster locator, online analysis and visualization of gene clustering. Bioinformatics **34**, 3377–3379 (2018). 29701747 10.1093/bioinformatics/bty336

[56] S. T. Kosak , Coordinate gene regulation during hematopoiesis is related to genomic organization. PLoS Biol. **5**, e309 (2007). 18031200 10.1371/journal.pbio.0050309 PMC2080650

[57] J. A. Guerrero-Martínez, M. Ceballos-Chávez, F. Koehler, S. Peiró, J. C. Reyes, TGFβ promotes widespread enhancer chromatin opening and operates on genomic regulatory domains. Nat. Commun. **11**, 6196 (2020). 33273453 10.1038/s41467-020-19877-5 PMC7713251

[58] Y. Chien , Control of the senescence-associated secretory phenotype by NF-κB promotes senescence and enhances chemosensitivity. Genes Dev. **25**, 2125–2136 (2011). 21979375 10.1101/gad.17276711 PMC3205583

[59] J. S. Tilstra , NF-κB inhibition delays DNA damage–induced senescence and aging in mice. J. Clin. Invest. **122**, 2601–2612 (2012). 22706308 10.1172/JCI45785 PMC3386805

[60] J. Massagué, TGFβ signalling in context. Nat. Rev. Mol. Cell Biol. **13**, 616–630 (2012). 22992590 10.1038/nrm3434 PMC4027049

[61] M. Namwanje, C. W. Brown, Activins and inhibins: Roles in development, physiology, and disease. Cold Spring Harb. Perspect. Biol. **8**, a021881 (2016). 27328872 10.1101/cshperspect.a021881 PMC4930927

[62] M. Fane, L. Harris, A. G. Smith, M. Piper, Nuclear factor one transcription factors as epigenetic regulators in cancer. Int. J. Cancer **140**, 2634–2641 (2017). 28076901 10.1002/ijc.30603

[63] L. Harris, L. A. Genovesi, R. M. Gronostajski, B. J. Wainwright, M. Piper, Nuclear factor one transcription factors: Divergent functions in developmental versus adult stem cell populations. Dev. Dyn. **244**, 227–238 (2015). 25156673 10.1002/dvdy.24182 PMC4563865

[64] A. R. Konopka, D. W. Lamming; RAP PAC Investigators, EVERLAST Investigators, Blazing a trail for the clinical use of rapamycin as a geroprotecTOR. Geroscience **45**, 2769–2783 (2023). 37801202 10.1007/s11357-023-00935-x PMC10643772

[65] R. Selvarani, S. Mohammed, A. Richardson, Effect of rapamycin on aging and age-related diseases-past and future. Geroscience **43**, 1135–1158 (2021). 33037985 10.1007/s11357-020-00274-1 PMC8190242

[66] E. K. Quarles, P. S. Rabinovitch, Transient and late-life rapamycin for healthspan extension. Aging **12**, 4050–4051 (2020). 32167485 10.18632/aging.102947 PMC7093168

[67] R. Stefancsik, S. Sarkar, Relationship between the DNA binding domains of SMAD and NFI/CTF transcription factors defines a new superfamily of genes. DNA Seq. **14**, 233–239 (2003). 14631647 10.1080/1085566031000141126

[68] U. Kruse, A. E. Sippel, Transcription factor nuclear factor I proteins form stable homo- and heterodimers. FEBS Lett. **348**, 46–50 (1994). 8026582 10.1016/0014-5793(94)00585-0

[69] M. Zenker , Variants in nuclear factor I genes influence growth and development. Am. J. Med. Genet. C Semin. Med. Genet. **181**, 611–626 (2019). 31730271 10.1002/ajmg.c.31747

[70] B.-H. Kim, K. Nho, J.-M. Lee; Alzheimer’s Disease Neuroimaging Initiative, Genome-wide association study identifies susceptibility loci of brain atrophy to NFIA and ST18 in Alzheimer’s disease. Neurobiol. Aging **102**, 200.e1–200.e11 (2021). 10.1016/j.neurobiolaging.2021.01.021 33640202

[71] C. Kang , The DNA damage response induces inflammation and senescence by inhibiting autophagy of GATA4. Science **349**, aaa5612 (2015). 26404840 10.1126/science.aaa5612 PMC4942138

[72] K. Tominaga, H. I. Suzuki, TGF-β signaling in cellular senescence and aging-related pathology. Int. J. Mol. Sci. **20**, 5002 (2019). 31658594 10.3390/ijms20205002 PMC6834140

[73] J. Massagué, D. Sheppard, TGF-β signaling in health and disease. Cell **186**, 4007–4037 (2023). 37714133 10.1016/j.cell.2023.07.036 PMC10772989

[74] J. C. Acosta , A complex secretory program orchestrated by the inflammasome controls paracrine senescence. Nat. Cell Biol. **15**, 978–990 (2013). 23770676 10.1038/ncb2784 PMC3732483

[75] M. Antsiferova , Mast cells are dispensable for normal and activin-promoted wound healing and skin carcinogenesis. J. Immunol. **191**, 6147–6155 (2013). 24227781 10.4049/jimmunol.1301350

[76] H. Bai, P. Kang, A. M. Hernandez, M. Tatar, Activin signaling targeted by insulin/dFOXO regulates aging and muscle proteostasis in Drosophila. PLoS Genet. **9**, e1003941 (2013). 24244197 10.1371/journal.pgen.1003941 PMC3820802

[77] K. Chang , TGFB-INHB/activin signaling regulates age-dependent autophagy and cardiac health through inhibition of MTORC2. Autophagy **16**, 1807–1822 (2020). 31884871 10.1080/15548627.2019.1704117 PMC8386626

[78] S. Haridoss , Activin A is a prominent autocrine regulator of hepatocyte growth arrest. Hepatol. Commun. **1**, 852–870 (2017). 29404498 10.1002/hep4.1106 PMC5721463

[79] C.-S. Kuo , Increased activin A levels in prediabetes and association with carotid intima-media thickness: A cross-sectional analysis from I-Lan Longitudinal Aging Study. Sci. Rep. **8**, 1–9 (2018). 29967428 10.1038/s41598-018-27795-2 PMC6028626

[80] E. Latres , Activin A more prominently regulates muscle mass in primates than does GDF8. Nat. Commun. **8**, 15153 (2017). 28452368 10.1038/ncomms15153 PMC5414365

[81] H. A. Loomans, C. D. Andl, Intertwining of activin A and TGFβ signaling: Dual roles in cancer progression and cancer cell invasion. Cancers **7**, 70–91 (2014). 25560921 10.3390/cancers7010070 PMC4381251

[82] L.-N. Peng , Association between serum activin A and metabolic syndrome in older adults: Potential of activin A as a biomarker of cardiometabolic disease. Exp. Gerontol. **111**, 197–202 (2018). 30071284 10.1016/j.exger.2018.07.020

[83] J. D. Roh , Activin type II receptor signaling in cardiac aging and heart failure. Sci. Transl. Med. **11**, eaau8680 (2019). 30842316 10.1126/scitranslmed.aau8680 PMC7124007

[84] Z. Deng , TGF-β signaling in health, disease, and therapeutics. Signal Transduct. Target. Ther. **9**, 61 (2024). 38514615 10.1038/s41392-024-01764-w PMC10958066

[85] J. J. Tao , First-in-human phase I study of the activin A inhibitor, STM 434, in patients with granulosa cell ovarian cancer and other advanced solid tumors. Clin. Cancer Res. **25**, 5458–5465 (2019). 31068369 10.1158/1078-0432.CCR-19-1065 PMC7899078

[86] P. Xu, J. Liu, R. Derynck, Post-translational regulation of TGF-β receptor and Smad signaling. FEBS Lett. **586**, 1871–1884 (2012). 22617150 10.1016/j.febslet.2012.05.010 PMC4240271

[87] M. J. Friedman, T. Wagner, H. Lee, M. G. Rosenfeld, S. Oh, Enhancer-promoter specificity in gene transcription: Molecular mechanisms and disease associations. Exp. Mol. Med. **56**, 772–787 (2024). 38658702 10.1038/s12276-024-01233-y PMC11058250

[88] J.-P. Vert, N. Foveau, C. Lajaunie, Y. Vandenbrouck, An accurate and interpretable model for siRNA efficacy prediction. BMC Bioinformatics **7**, 520 (2006). 17137497 10.1186/1471-2105-7-520 PMC1698581

[89] W. Li , Functional roles of enhancer RNAs for oestrogen-dependent transcriptional activation. Nature **498**, 516–520 (2013). 23728302 10.1038/nature12210 PMC3718886

[90] S. Heinz , Simple combinations of lineage-determining transcription factors prime cis-regulatory elements required for macrophage and B cell identities. Mol. Cell **38**, 576–589 (2010). 20513432 10.1016/j.molcel.2010.05.004 PMC2898526

