# Supplementary material for: Ligand-specific regulation of a binary enhancer code dictating cellular senescence
Source: Proc Natl Acad Sci U S A. 2025 Jun 10;122(24):e2506321122. doi: 10.1073/pnas.2506321122 (PMC12184664; doi:10.1073/pnas.2506321122)
Supplement: Supplementary file 1 — Appendix 01 (PDF) [file pnas.2506321122.sapp.pdf]

PNAS

Supporting Information for

Ligand-Specific Regulation of a Binary Enhancer Code Dictating Cellular Senescence

Thomas Suter[1,#]; Meyer J. Friedman[1,#]; Cagdas Tazearslan[2,#]; Daria Merkurjev[1]; Kenny Ohgi[1]; Dario Meluzzi[1]; Michael G. Rosenfeld[1,*,+]; Yousin Suh[2, 3, 4,*]

[1] Cellular and Molecular Medicine, Department of Medicine, University of California San Diego, La Jolla, CA, USA
[2] Department of Genetics, Albert Einstein College of Medicine, Bronx, NY, USA
[3] Department of Obstetrics and Gynecology, Columbia University Irving Medical Center, New York, NY, USA
[4] Department of Genetics and Development, Columbia University Irving Medical Center, New York, NY, USA

[#] equal contributions
[+] Lead Contact

[*] To whom correspondence should be addressed:

Yousin Suh: ys3214@cumc.columbia.edu;
Michael G. Rosenfeld: mrosenfeld@health.ucsd.edu

This PDF file includes:

Extended methods
Figures S1 to S4
Tables S1 to S5
SI References

Supporting Information Text

Extended methods

Tissue culture
IMR90 fibroblasts cultured prior to PD34 after initial thawing and showing a doubling time of less than 2 days were deemed "early passage," while IMR90 cells collected after PD52 that failed to double within 1 week were considered to have reached replicative senescence.

For oncogene-induced senescence (OIS) experiments, IMR90 cells were transduced at PD30 with either pBABE-puro or pBABE-puro H-Ras V12 retrovirus. Selection with 1ug/ml of puromycin was initiated 4 days post transduction and maintained until harvesting at 14 days post transduction. OIS was confirmed by beta-galactosidase staining of cells prior to harvesting.

Short-term shRNA experiments were performed by transducing either senescent (>PD60) or HPGA rapamycin-maintained BJ fibroblasts with pLKO.1 lentivirus. Lentiviral-expressing cells were selected with 0.6 ug/ml puromycin starting 4 days after transduction and continuing until 14 days after transduction, at which point cells were harvested. The same protocol was used for enhancer knockdown experiments in senescent BJ fibroblasts with pLKO.1 constructs.

For HPGA rapamycin-maintained BJ fibroblasts, both rapamycin and puromycin (0.6 ug/ml) were added to the culture media until 14 days after transduction, at which point cells were split into 2 plates for rapamycin maintenance or withdrawal as described above.

For long-term shRNA experiments, early passage (<PD30) BJ fibroblasts were transduced with pLKO.1, maintained under selection with 0.6ug/ml puromycin while expanding for 2 weeks, and then cryopreserved upon harvesting. After thawing, stable lines were grown continuously until reaching senescence, and their population doublings were recorded over time. Both early passage and senescent stable knockdown lines were harvested for further experiments.

Construction and acquisition of shRNA vectors
Like enhancer knockdown constructs, shRNA vectors targeting p65 employed the pLKO.1 backbone. The pLKO.1-TRC cloning vector (Addgene Plasmid #10878) was digested with AgeI and EcoRI restriction enzymes. Oligonucleotides complementary to the target sequence (*SI Appendix*, **Table S3**) containing overhangs compatible with AgeI and EcoRI restriction sites were synthesized, annealed, and ligated to the digested vector. shRNA constructs targeting NFIA, NFIB, NFIC, NFIX, SMAD2, and SMAD3 were purchased from the Albert Einstein College of Medicine shRNA Core Facility (*SI Appendix*, **Table S4**).

Virus production and transduction conditions
Lentivirus and retrovirus for OIS experiments were produced by transfection of packaging plasmids into 293T cells (ATCC® CRL-3216) at ~80% confluency. Lipofectamine 2000 (Thermo Fisher 12566014) was used for all transfections, following the manufacturer's protocol, with the ratios of plasmids and Lipofectamine 2000 reagent listed below.

Retrovirus for OIS experiments was produced by combining a 7.5 : 7.5 ug ratio of pHCMV-AmphoEnv (Addgene 15799) packaging plasmid and pBABE vector (either pBABE-puro (Addgene 1764) or pBABE-puro H-Ras V12 (Addgene 9051)) with 51ul of Lipofectamine 2000 in a 10cm dish. Lentivirus for use in shRNA knockdown experiments was generated by transfecting a 0.625 : 1.875 : 2.5 ug ratio of MD2.G envelope plasmid: psPAX2 packaging plasmid: pLKO.1 shRNA-expressing transfer plasmid with 17ul Lipofectamine 2000 in a single well of a 6-well plate. Media was changed 6 hours post transfection, and the virus-containing media was collected at 48 and 72

hours post transfection. Virus-containing media was centrifuged at 600g for 5 min, and the viral supernatant was collected and stored at 4°C prior to transduction.

Retroviral transduction was performed on actively growing IMR90 and BJ fibroblasts at ~50% confluency. Lentiviral transduction was performed on both early passage and senescent BJ fibroblasts at ~50% confluency. For pBABE transductions for OIS experiments, viral supernatants were mixed 1:1 with fresh culture media. pLKO.1 stable knockdown lines were generated using a 48 ul : 1952 ul ratio of pLKO.1 viral supernatant with fresh culture media. All transduction mixtures were added to cells overnight with polybrene (8ug/ml final concentration). OIS and control cells were maintained in 1ug/ml puromycin starting 4 days after transduction. Knockdown cells were maintained in 0.6ug/ml puromycin starting 4 days after transduction. All shRNA knockdowns were validated via qPCR to elicit >75% reduction in mRNA targets.

Reverse transcription and qPCR
Reverse transcription for quantification of transcripts was done with SuperScript III Reverse Transcriptase (ThermoFisher Scientific-18080044) as per manufacturer's instructions. qPCR was then performed on the resultant cDNA using VeriQuest SYBR Green qPCR Master Mix (2x) with Rox (ThermoFisher Scientific-C-75690) following manufacturer's instructions. Primers for qPCR quantification of enhancer knockdown are listed in **Table S5** (*SI Appendix*). Statistics for qPCR data represent two-tailed t-tests between control and experimental conditions, with 3 or more biological replicates per condition. All replicates with healthy cells were included (i.e., adherent cells without evidence of cell death that were not sparsely distributed).

Microscopy
Imaging of SA-β-gal staining was performed with either the Keyence BZ-X710 in 20x color mode or the Zeiss Axiovert 200M. Imaging of morphology of rapamycin withdrawn cells after sh-p65 or sh-control knockdown was done with the Perkin Elmer UltraView Vox Spinning Disk Confocal at 20X.

SA-β-gal staining
β-Gal staining of senescent cells was performed using the Senescence β-Galactosidase Staining Kit (Cell Signaling #9860S). In brief, BJ or IMR90 fibroblasts were incubated in Fixative solution for 15 minutes at room temperature. After washing, cells were incubated in β-Galactosidase Staining Solution overnight at 37°C in a dry incubator. Samples were imaged by bright-field microscopy to assess positive (blue) SA-β-gal staining of cells.

ChIP-seq
Samples for histone modification ChIPs were fixed in 1% formaldehyde for 10 minutes at room temperature. Samples for transcription factor (NFI, p65, and SMAD2/3) ChIPs were double crosslinked in 2mM DSG for 45 minutes at room temperature followed by 10-minute fixation with 1% formaldehyde. After neutralization with 0.125M glycine, nuclei were isolated and resuspended in nuclear lysis buffer (50mM Tris/HCl pH7.4, 1% SDS, 10mM EDTA, 1x cOmplete Protease Inhibitor Cocktail (Sigma 11836145001)), and then sonicated using the Bioruptor (Diagenode). The concentration of chromatin in each sample was determined using the Qubit Fluorometer (Thermo Fisher), and samples were diluted 1:10 in dilution buffer (20mM Tris/HCl pH7.4, 100mM NaCl, 0.5% Triton-X-100, 2mM EDTA, 1x cOmplete Protease Inhibitor Cocktail). For each histone or transcription factor ChIP assay, 20-50 ug of chromatin from each sample was diluted 1:10 for a final volume of 0.5 mL. Antibodies were incubated with each sample overnight.

Antibody:chromatin complexes were immunoprecipitated as described using 20 uL Protein G Dynabeads (Thermo Fisher). Histone modification ChIPs were washed with the following buffers/conditions in the indicated order: 2x with WB1 (20mM Tris/HCl pH7.4, 150mM NaCl, 1% Triton-X-100, 0.1% SDS, 2mM EDTA), 1x with WB2 (20mM Tris/HCl pH7.4, 500mM NaCl, 1% Triton-X-100, 2mM EDTA), 1x with WB3 (10mM Tris/HCl pH7.4, 250mM LiCl, 1% NP-40, 1% Deoxycholate, 1mM EDTA), and 2x with TE (10mM Tris/HCl pH 8.0, 1mM EDTA). Transcription factor ChIPs were washed with the following buffers in the indicated order: 2x with WB1 (20mM

Tris/HCl pH7.4, 150mM NaCl, 1% Triton-X-100, 2mM EDTA), 1x with WB2 (20mM Tris/HCl pH7.4, 300mM NaCl, 1% Triton-X-100, 2mM EDTA), 1x with WB3 (20mM Tris/HCl pH7.4, 400mM NaCl, 1% Triton-X-100, 2mM EDTA), and 2x with TE (10mM Tris/HCl pH 8.0, 1mM EDTA). After washing steps, samples were eluted in EB (1% SDS, 0.1M $NaHCO_3$) at 65 °C, decrosslinked overnight at 65 °C, and then purified using the QIAquick PCR Purification Kit (QIAGEN). Finally, samples were sonicated for an additional 10 minutes to ensure the majority of purified chromatin was 100-300bp.

GRO-seq

GRO-seq was performed as described previously (1). In brief, approximately 10 million BJ fibroblasts (either senescent or quiescent) were used per experimental condition. Cells were incubated for 5 minutes on ice in swelling buffer (10mM Tris-Cl pH7.5, 2mM MgCl2, 3mM CaCl2), lysed in lysis buffer (swelling buffer with 0.5% IGEPAL and 10% glycerol), and finally re-suspended in 100ul freezing buffer (50mM Tris-Cl pH8.3, 40% glycerol, 5mM MgCl2, 0.1mM EDTA). The run-on reaction was then performed by mixing the re-suspended nuclei with an equal volume of run-on reaction buffer (10mM Tris-Cl pH 8.0, 5mM MgCl2, 1mM DTT, 300mM KCl, 20 units of Superase-In, 1% sarkosyl, 500µM ATP, 500µM GTP, 500µM Br-UTP, and 2µM CTP) and incubating tubes for 5 min in a 30°C water bath. Nuclear run-on RNA (NRO-RNA) was extracted with TRIzol LS reagent (Invitrogen), as per the manufacturer's instructions. NRO-RNA was hydrolyzed to ~300bp fragments via 35-minute incubation on ice in 0.2M NaOH, which was followed by treatment with DNase I and Antarctic phosphatase. Br-UTP labeled NRO-RNA was isolated by incubation with anti-BrdU agarose beads (Santa Cruz Biotech) in binding buffer (0.5×SSPE, 1mM EDTA, 0.05% Tween 20) on a rotator at 4°C for 3h. T4 PNK (NEB) was then used to end-repair isolated NRO-RNA.

RNA fragments were incubated with poly-A polymerase (NEB) for 30 min at 37°C, after which reverse transcription was performed using Superscript III (Invitrogen) and the oNTI223 primer (**Table S5,** *SI Appendix*). cDNA products were run on a 10% polyacrylamide TBE-urea gel, and products between 100-500bp were excised and recovered by gel extraction. First strand cDNA was circularized via CircLigase (Epicentre) and then re-linearized by treatment with Ape1 (NEB). The re-linearized cDNA was then size selected on a TBE gel, and fragments between ~120-320 bp were recovered via gel extraction. Finally, this cDNA was PCR amplified using Phusion High-Fidelity enzyme (NEB) with oNTI200 ID1-6 (each sample was amplified with a different ID# primer to impart a distinct barcode) and oNTI201 (**Table S5,** *SI Appendix*). Samples were sequenced on the HiSeq 2500 system with single read runs of 50 cycles.

Processing of deep sequencing data

FASTQC was generally performed, and all replicates passing FASTQC were processed and included in analyses. All deep-seq reads were aligned to the hg18 assembly. ChIP-seq data were mapped using STAR-aligner with parameters adjusted to ignore spliced alignments. Aligned reads were processed for downstream analysis using the HOMER (2) "makeTagDirectory" program, filtering for uniquely mapped tags and selecting at most one sequencing read per position per length of read. Peaks for H3K27ac and $H3K4me^2$ were called using the HOMER "findPeaks" program with the "-histone" style parameter in order to identify broader peaks of at least 500bp and stitch together peaks of less than 1000bp. Transcription factor peaks were determined using the HOMER "findPeaks" program with the "-factor" style parameter to select peaks with a fixed width per experiment based on autocorrelation analysis estimates made during the construction of the tag directories.

To assemble a complete collection of enhancers in the progression to replicative senescence, we first identified all peaks from H3K27ac and $H3K4me^2$ ChIP-seq assays in EPQ, intermediate quiescent, and old BJ fibroblasts. The coordinates of these individual enhancer lists were merged using the HOMER "mergePeaks" program. Enhancers within 200bp of each other were combined into a single enhancer defined by their outer most coordinates (i.e. creating the widest possible enhancer). Enhancers were then filtered by removing those with less than 10 normalized tags for both H3K27ac and $H3K4me^2$ in a single experimental condition.

Quantitation of ChIP-seq signal at enhancers was done using the "annotatePeaks.pl" program in HOMER with the default normalization of 10 million tags. Meta-analyses and heatmap analyses of ChIP-seq data were also performed with the HOMER "annotatePeaks.pl" program, using the respective subset of genomic coordinates listed in each figure. Fold changes in histone ChIP-seq tag density were normalized by calculating the $\log_2$ fold change in tag density between two conditions at all 55,027 enhancers, determining the median fold change between two experiments over these 55,027 enhancers, and then subtracting the fold change of each enhancer by this amount. The median-normalized fold changes of enhancer histone marks are presented as box plots.

RNA-seq and GRO-seq data were mapped using STAR-aligner with parameters adjusted to account for spliced alignment. Aligned reads were processed for downstream analysis using the HOMER "makeTagDirectory" program, filtering for uniquely mapped tags and selecting at most one sequencing read per position per length of read. A preliminary set of transcript coordinates was generated by selecting the coordinates of the longest isoform for each gene. Our RNA-seq and GRO-seq datasets in EPQ, senescent, HPGA rapamycin withdrawal, and HPGA rapamycin-maintained conditions were then quantified over these preliminary transcript coordinates using the HOMER "annotatePeaks.pl" program. Transcripts with less than an average of at least 7 tags in both preliminary RNA-seq and GRO-seq experiments from a combination of EPQ, senescent, HPGA rapamycin-maintained and -withdrawn conditions were removed by filtering, leaving 14493 transcripts above our expression threshold for downstream analysis.

Processed RNA and GRO-seq datasets were quantified over this final transcript list of 14493 genes using the "annotatePeaks.pl" program of HOMER. A $\log_2$ normalized fold change in tag density was determined between each condition over all 14493 genes, which was then median normalized for each comparison so that the median $\log_2$ fold change in tag density over all 14493 transcripts between two experiments would be 0 (similar to normalization of enhancer mark fold changes described in Methods). Box plots represent the distribution of these median-normalized fold changes for various listed subsets of the 14493 gene transcripts. Figures showing fold changes in transcription refer to an averaging of all replicates of RNA-seq and GRO-seq performed in the indicated condition.

Processed deep-seq data were visualized via the University of California, Santa Cruz (UCSC) genome browser after preparation with HOMER. Motif analysis of enhancer and ChIP-seq peaks was performed with the HOMER "findMotifs.pl" program, using the coordinates of the indicated enhancer set as input for *de novo* assessment. Gene Ontology (GO) analysis was done with the web-based Metascape application (3). Gene Set Enrichment Analysis (GSEA) was performed using GSEA software as described (4, 5). Expressed genes were ranked based on their transcriptional fold change in the condition noted on the x-axis, and the query gene set indicated in the figure caption was input in order to determine its distribution relative to the ranked gene list.

ATAC-seq procedure and data analysis

~50,000 BJ fibroblasts (senescent or quiescent via contact inhibition) were collected via trypsinization, washed in PBS on ice, resuspended in 200uL ice-cold ATAC-seq lysis buffer (10mM Tris-HCl pH 7.5, 10mM NaCl, 3mM $MgCl_2$, 0.1% Igepal CA-630) by gently pipetting up and down 10 times, and immediately centrifuged for 10 minutes at 1000g and 4°C. The tagmentation reaction was performed using the Nextera DNA Library Prep Kit (Illumina-FC-121-1030). The pellet was resuspended in 50uL 1x TD buffer containing 2.5uL Tn5 Transposase. Following incubation at 37°C for 30 minutes, DNA was purified using the MiniElute Reaction Cleanup Kit (Qiagen-28204). Tagmented DNA was prepared for deep sequencing with the KAPA Real-Time Library amplification kit (KAPA/Roche-KK2701), as per manufacturer's instructions, using Illumina Nextera primers. Individual sample reactions were removed separately from the qPCR machine when their amplification reached that of Standard 2, which was typically after approximately 10 cycles. The amplified samples were size selected for 150-800bp fragments with AmPureXP beads (Beckman-A63880) and then sequenced using the Illumina Nextera sequencing primer on the HiSeq 2500 with 100 cycle single-read runs.

ATAC-seq data were mapped using STAR-aligner, and aligned reads were processed for downstream analysis using the HOMER "makeTagDirectory" program, filtering for tags that were uniquely mapped and selecting no more than one sequencing read per position per length of read. For the generation of box plots and meta-analyses, tag counts and tag density plots from each experiment were normalized to the total number of unique tag positions to account for differences in sequencing depth, PCR duplicates, and contaminating mitochondrial DNA. This normalization strategy was validated by confirming similar meta-analysis tag density distributions in each experiment over genes unchanged in senescence (**Fig. 3E**).

Determination of gene expression sets and subsets
Genes classified as up- or down-regulated in either senescence or rapamycin withdrawal (S_{GAINED}, S_{LOST}, and RW_{GAINED}) were at least 1 standard deviation beyond a 1.5-fold change in expression when comparing multiple replicates of RNA-seq and GRO-seq for these conditions. Only genes that exceeded an expression threshold of 9 tags per million (>9 TPM) were considered. Paired samples consisting of 3 RNA-seq replicates and a single GRO-seq experiment for EPQ vs senescent BJ fibroblasts were used to determine S_{GAINED} and S_{LOST} genes, comprising 639 and 598 genes, respectively. Similarly, 8 RNA-seq and 2 GRO-seq paired replicates of HPGA rapamycin-maintained and withdrawn BJ fibroblasts were used to assign 252 RW_{GAINED} genes. The S_{GAINED}-SASP and S_{GAINED}-PROLIF subsets consisted of the 150 or 300 genes within the S_{GAINED} set that were either most or least induced, respectively, upon rapamycin withdrawal from HPGA BJs (using an average of the 10 aforementioned rapamycin withdrawal paired replicates). Unless otherwise indicated, the 150 gene subsets were used, while the 300 gene subsets were typically reserved for GO analysis in order to increase statistical confidence with a larger cohort of genes.

***In-situ* Hi-C**
In-situ Hi-C was performed as described in Rao et al., 2014 (6). In brief, approximately 1 million BJ fibroblasts (senescent or quiescent via contact inhibition) were collected per experimental condition, and isolated nuclei were treated overnight with MboI restriction enzyme at 37°C while rotating at 8 RPM. Nuclei were then treated with Klenow in the presence of biotin-CTP in order to both blunt-end and biotin label the digested chromatin. Next, in-situ ligation was performed by treating nuclei with HC T4 DNA ligase (Enzymatics). Ligated chromatin was isolated by incubating nuclei in SDS solution containing proteinase K followed by phenol/chloroform/isoamyl alcohol extraction. Isolated chromatin was sheared to ~300bp by Covaris sonication based on the manufacturer's protocol. Fragments >400bp were removed via size selection with AmPure XP beads (Beckman-A63880). Purified chromatin fragments were bound to MyOne Streptavidin T1 Dynabeads (ThermoFisher Scientific-65601), blunted with Klenow, and poly-A tailed via Exo-Klenow treatment. Illumina TruSeq adapters were ligated to the bead-attached chromatin fragments with HC T4 DNA ligase. Libraries were PCR amplified using NEBNext High Fidelity 2x PCR Master Mix (NEB-M0541S) and Illumina TruSeq amplification primers. Amplified libraries were size-selected with AmPure beads and sequenced using the HiSeq 4000 with 50 cycle paired-end runs.

Hi-C data analysis

Construction of contact matrices
Reads pairs obtained by sequencing the Hi-C libraries were mapped to the human reference genome assembly hg18 using bowtie2 version 2.3.0 with the following parameters: --end-to-end --very-sensitive -L 30 --score-min L,-0.6,-0.2 --mp 6,2. For each pair, each 50-base read was mapped independently. Reads aligning to multiple genomic locations were discarded. Reads that could not be aligned to any genomic location were further processed as follows. The 8-base sequence expected for the junction resulting from ligation of the blunt ends of two Hi-C fragments was searched within the read sequence, allowing for a single base mismatch. If the searched junction sequence was found, then the read sequence was split at the midpoint of the junction and the resulting sub-sequences were mapped independently to the reference genome. If the junction

sequence was not found, then the read sequence was processed in a manner similar to the iterative mapping procedure of Imakaev et al (7). Sub-sequences starting from each end of the read were iteratively extracted and independently mapped to the reference genome. The initial length of each sub-sequence was 16 bases and was incremented by 5 bases until the sub-sequence did not align to multiple genomic locations. All read sequences and sub-sequences that successfully aligned to unique genomic locations were then grouped by read pair of origin. Groups of size greater than one were analyzed to infer pairs of read sequences, or sub-sequences, that likely resulted from pairs of crosslinked Hi-C fragments. For each inferred sequence pair, the two alignment coordinates were used to filter out duplicated pairs, likely resulting from PCR amplification, and spurious intra-chromosomal interactions, likely resulting from dangling ends and self-ligation products, as described by Jin F et al (8). Specifically, sequence pairs aligned to genomic locations closer than 500 bp were classified as "samestrand" and "outward" pairs and hence discarded. The remaining aligned sequence pairs were used to generate symmetric matrices of raw contacts counts for all possible pairs of genomic bins, with one matrix per chromosome and 250 kb per bin. The contact matrices were pruned by removing rows and columns that contained too many zero entries in all of the samples considered. The minimum acceptable number of non-zero entries was arbitrarily chosen to be 25% of the median number of non-zero entries in each row. To remove experimental biases, the resulting contact matrices were processed with the iterative matrix-balancing procedure of Imakaev et al (7).

Calculation of A/B compartment profiles

To obtain profiles of A/B compartment for each chromosome, the matrix of unbiased contact counts for a given chromosome was converted into a z-score matrix by calculating local mean and standard deviation within a window of 41 elements (10.25 Mb) along the diagonal through each element of the unbiased contact count matrix. The main and first adjacent diagonals of the z-score matrix were set to zero, to avoid excessive influence from the large values in those diagonals. A matrix of Pearson correlation coefficients was then computed from all possible pairs of rows and columns in the z-score matrix. The first principal component of the Pearson correlation matrix (i.e., the first eigenvector of the covariance matrix) was taken to be the desired A/B compartment profile for the given chromosome. The sign of such profile was adjusted to produce a positive covariance with a profile of GC density collected from the reference sequence over the same chromosome. Therefore, positive values of the A/B compartment profile correspond to A compartments, which are thought to be enriched for gene promoters and thus have high GC content.

Comparison of A/B compartment profiles

To identify statistically significant changes in A/B compartment profiles when compared between different experimental conditions, multiple experimental replicates should ideally be considered for each condition. In the absence of such replicates, simulation was used to generate artificial replicates of A/B compartment profiles. For each chromosome, an average binomial contact probability was calculated for each pair of interacting genomic bins by summing the raw contact matrices from all experimental conditions and then dividing the resulting matrix by the total sum of counts in that matrix. The matrix of contact probabilities was multiplied element-wise by the raw contact count matrix for each given experimental condition to obtain a matrix of mean contact counts for that condition. The mean contact counts were used to fill a new matrix with Poisson-distributed random numbers. The latter matrix was then processed in a manner identical to the corresponding experimental contact count matrix, thus yielding a simulated A/B compartment profile for the given chromosome and experimental condition. Three such profiles were generated for each chromosome and experimental condition, each time using a different seed of the random number generator. The sample standard deviation was calculated across those profiles for each genomic bin of 250 kb. To compare the experimental A/B compartment profiles for two given conditions, a t-test was performed at each genomic bin by applying the function ttest_ind_from_stats provided by the Python package scipy.stats. In lieu of the sample means and sample standard deviations expected by this function, the values of the two compared experimental profiles and the standard deviations of the corresponding simulated profiles were passed to that function. The p-values thereby obtained for all bins across the genome were adjusted for multiple testing by following the procedure of Benjamini and Hochberg (9).

Gene cluster analysis
The presence and distribution of gene clusters in the senescent up-regulated (639 S_{GAINED}), senscent down-regulated (598 S_{LOST}), and rapamycin withdrawal-gained (252 RW_{GAINED}) gene sets was determined using the Cluster Locator tool web interface (10). As an additional control, a set of 650 expressed genes was randomly selected from the collection of gene transcripts that exceeded a minimum expression threshold in BJ fibroblasts and were unchanged in replicative senescence.

Analysis of publicly available datasets
The following publicly available RNA-seq datasets were acquired from the GEO database:
SRR3573989, SRR3573990, SRR3573991, SRR3573992, SRR3573995, SRR3573996, SRR6277486, SRR6277487, SRR8676836, SRR8676837, SRR3185780, SRR3185782, SRR3185784, SRR3185786, SRR3185788, SRR3185790, SRR3185792, SRR3185794, SRR3185796, SRR2378601, SRR2378602, SRR2378604, SRR2378605, SRR5680551, SRR5680552, SRR5680557, SRR5680558, SRR5680563, SRR5680564, SRR5680569, SRR5680570, SRR5680575, SRR5680576, SRR5680581, SRR5680582, SRR5680587, SRR5680588, SRR5680593, SRR5680594, SRR6153877, SRR6153878, SRR6153879, SRR6153880, SRR6153881, SRR6153882, SRR6153883, SRR6153884, SRR6153885, SRR6153886, SRR6153887, SRR6153888, SRR6153889, SRR6153890, SRR6153891, SRR6153892, SRR8422990, SRR8422991, SRR8422992, SRR8422994, SRR8422995, SRR8422996, SRR8423006, SRR8423007, SRR8423010, SRR8423011, SRR8482072, SRR8482073, SRR8482074, SRR8482075, SRR8482076, SRR8482077, SRR9718626, SRR9718627, SRR9718628, SRR9718629, SRR9718630, SRR9718631, SRR9718632, SRR9718633, SRR9718634, SRR9718635, SRR9718636, SRR9718637, SRR9718638, SRR9718639, SRR9718640, SRR9718641, SRR10034209, SRR10034210, SRR10034213, SRR10034214, SRR10034217, SRR10034218, SRR7537409, SRR7537410, SRR7537411, SRR7537418, SRR7537419, SRR7537420, SRR7537427, SRR7537428, SRR7537429.

All datasets were derived from human cells and involved samples treated with Activin A, TGF-β1, TGF-β2, or vehicle control. Samples were processed in the same manner as RNA-seq datasets generated in this study, as described above. After alignment and gene annotation, RNA-seq tag counts in ligand-treated samples were divided by the tag counts of their respective control samples, median normalized over the entire population of genes, and the fold changes for *INHBA*, *TGFB1*, and *TGFB2* in each sample/treatment were determined.

GREAT meta-analysis
Enhancer sets used for GREAT analysis were defined by Activin A vs TGF-β2 selective SMAD2/3 binding in response to Activin A, TGF-β2, Inhibin A, TGF-β2+Inhibin A, or control treatments in either senescent or rapamycin-withdrawn HPGA cells. Enhancer sets featuring higher SMAD2/3 binding in an Activin A-associated condition were paired with enhancer sets showing higher SMAD2/3 binding in a TGF-β2-associated condition. The paired enhancer sets are listed in the **Table S2 (***SI Appendix*).

BED-formatted enhancer coordinates for each set were uploaded at the GREAT web-interface (http://great.stanford.edu/public/html/). GREAT analysis was performed under the hg38 assembly, after lifting over coordinates from hg18, using the whole genome as background and the Basal plus extension method default parameters (proximal: 5kb upstream, 1kb downstream plus Distal: up to 1000kb) for associating non-coding genomic regions with genes.

Meta-analysis of the GREAT output was performed by scoring the top 500 GO Biological Process results based on their Binomial Ranking, with the top-ranked result scored as 500 and the 500th-ranked result scored as 1. The top 500 GO Biological Process results were then filtered and categorized based on the semantic similarity of their descriptions to terms related to either proliferation regulation or SASP. Results whose descriptions included either “proliferation”, “cell cycle”, or “replication” were designated as proliferation-associated, whereas results whose

descriptions included “secretion”, “cytokine”, “inflammatory”, or “NF-kappaB” were classified as SASP-associated. The analysis in **Fig. S4F** (*SI Appendix*) directly plots these selected terms for the listed enhancer sets, colorized by their association with either proliferation (green) or SASP (red), with brightness reflecting the binomial rank (black meaning the term was not present in the top 500 GO Biological Process results from the GREAT analysis for that particular enhancer group).

The rank-scores of the semantically categorized GO Biological Process results were summated within each category, resulting in each enhancer set having a rank-score sum for the Proliferation category and for the SASP category. For each enhancer set, a Proliferation/SASP ratio was calculated by dividing its Proliferation category rank-score sum by its SASP category rank-score sum. Box plots showing the distribution of Prolif/SASP GREAT ratios for all enhancer sets included in **Table S2** (*SI Appendix*) are presented in **Fig. 4J**. The p-value for the comparison of Activin A- vs TGF-β2+Inhibin A-regulated enhancer sets was calculated using the Wilcoxon signed rank test.

GWAS/PheWAS analysis

GWAS analysis was performed using 104 different enhancer sets, each defined by unique criteria based on ChIP-seq data in this study. Each enhancer set was assessed across a combined 531 PheWAS and GWAS traits (11, 12) for significant enrichment (FDR<0.01) of trait-associated SNPs. Traits with SNPs enriched in the S_{GAINED} enhancer group are shown in **Fig. S1A** (*SI Appendix*).

Meta-analysis of the GWAS/PheWAS results was performed by associating 83 of the 104 enhancer sets with either proliferation or SASP/inflammation. These enhancer groups were scored based on our model’s prediction of the role of the criteria (i.e., cell state, culturing condition, treatment, etc) used to define them. Each of the 83 enhancer sets was then tested for enrichment of any GWAS/PheWAS trait SNPs. **Fig. S4G** (*SI Appendix*) shows the GWAS/PheWAS traits with significantly overrepresented SNPs in enhancer sets that are most strongly imbalanced towards either proliferation or SASP/inflammation in their scores.

A

Enhancer Group	GWAS Phenotype	Fisher p-value	FDR
S_{GAINED}	[gwas] chronic lymphocytic leukemia	1.77E-04	1.54E-03
S_{GAINED}	[gwas] coronary artery disease	1.58E-03	9.69E-03
S_{GAINED}	[gwas] Graves disease	7.75E-04	5.35E-03
S_{GAINED}	[gwas] multiple sclerosis	3.32E-04	2.62E-03
S_{GAINED}	[phecode 420.4] Other rheumatic heart disease	8.33E-06	1.03E-04

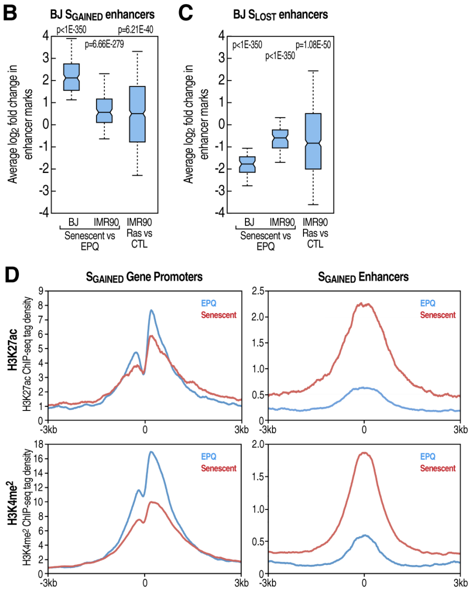

Suter et al. 2025, Fig. S1

Figure S1. Gained enhancers regulate induced transcriptional programs in cellular senescence. **A)** Analysis of S_{GAINED} enhancers for enrichment of SNPs linked to 531 GWAS and PheWAS traits. Only traits with enrichment FDR < 0.05 are shown. **B, C)** Box plots of change in H3K27ac and H3K4me^2 ChIP-seq tag density during BJ fibroblast replicative senescence, IMR90 replicative senescence, or IMR90 oncogene induced senescence (Ras vs CTL). Averaged fold change in tag density at **B)** S_{GAINED} or **C)** S_{LOST} enhancers is shown. **D)** Meta-analysis of normalized H3K27Ac (top) and H3K4me^2 (bottom) ChIP-seq tag density distributions in EPQ and replicatively senescent BJ fibroblasts, centered on the TSSs of S_{GAINED} genes (left) or on the midpoint of S_{GAINED} enhancer peaks (right).

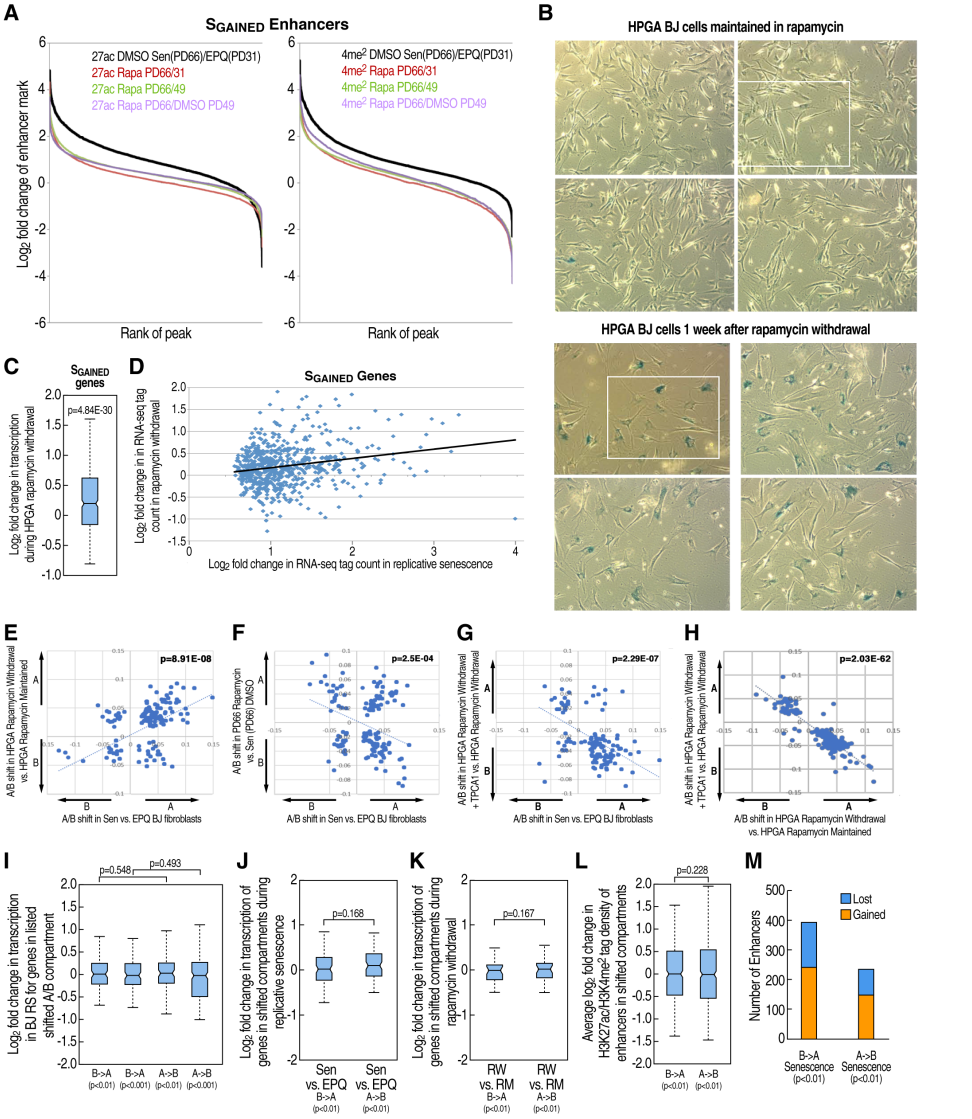

Suter et al. 2025, Fig. S2

Figure S2. Withdrawal of high-passage, growth-arrested (HPGA) cells from rapamycin maintenance selectively affects the SASP program of replicative senescence. **A)** Ranked fold change of ChIP-seq peak scores for H3K27ac (left) and H3K4me^2 (right) in indicated conditions at a modified set of Sgained enhancers. For these analyses, the SGAINED enhancer set was compiled from H3K27ac and H3K4me^2 ChIP-seq replicates independent from those plotted in the figure. Rapa, rapamycin; PD, population doubling; Sen, senescent. **B)** SA-β-gal staining of HPGA BJ fibroblasts maintained in 10nM rapamycin or withdrawn from rapamycin treatment for 1 week. Images for each condition represent different fields of view, and white squares correspond to insets presented in Fig. 2C. **C)** Box plot of fold change in transcription of SGAINED genes for HPGA rapamycin-withdrawn versus rapamycin-maintained cells. **D)** Scatter plot of fold change in transcription of SGAINED genes in HPGA rapamycin-withdrawal condition versus replicative

senescence. **E, F)** Analysis of *in situ* Hi-C demonstrating that senescence-associated A/B compartment changes are reduced by rapamycin. Each point represents a 500kb bin whose compartmental identity changed between the two conditions with $p < 0.01$. The x-axis value is the difference in A/B shift between EPQ and senescent cells (where positive values indicate a shift towards the A compartment during replicative senescence), while the y-axis value is the difference in A/B shift between **E)** HPGA rapamycin-maintained and (2 week) rapamycin-withdrawn cells (where positive values indicate a shift towards the A compartment in HPGA rapamycin-withdrawn cells; positive correlation p = 8.91 e-08) or **F)** Senescent vehicle (DMSO)-treated cells and cells of the same passage number maintained in rapamycin (negative correlation p = 2.5 e-04). **G, H)** Analysis of *in situ* Hi-C showing that senescence-associated A/B compartment changes are NF-κB dependent. The x-axis value is the difference in A/B shift between **G)** EPQ and senescent cells (where positive values indicate a shift towards the A compartment during replicative senescence) or **H)** HPGA rapamycin-maintained and (2 week) rapamycin-withdrawn cells. The y-axis value is the difference in A/B shift between HPGA rapamycin-withdrawn and TPCA-1-treated, (2 week) rapamycin-withdrawn cells. For negative correlation in (G), p=2.29 e-07; for negative correlation in (H), p=2.03 e-62. Reported p-values in E-H were Benjamini-Hochberg adjusted (see Extended methods, *SI Appendix*). **I)** Box plot of alterations in expression, as an average of $\log_2$ fold change in normalized RNA- and GRO-seq tag counts, for genes in shifted A/B compartment bins during replicative senescence. **J, K)** Box plots of alterations in expression, as an average of $\log_2$ fold change in normalized RNA- and GRO-seq tag counts, for genes in shifted A/B compartment bins during **J)** replicative senescence relative to quiescence (EPQ) or **K)** HPGA rapamycin withdrawal (RW) relative to rapamycin maintenance (RM). **L)** Box plot of alterations in chromatin marks, as an average of $\log_2$ fold change in normalized tag counts from duplicate H3K27ac and $H3K4me^2$ ChIP-seq experiments in replicative senescence, at enhancers located in bins with shifted A/B compartmentalization. **M)** Stacked bar graph quantifying gained and lost enhancers during replicative senescence in bins with significantly changed A/B compartmentalization.

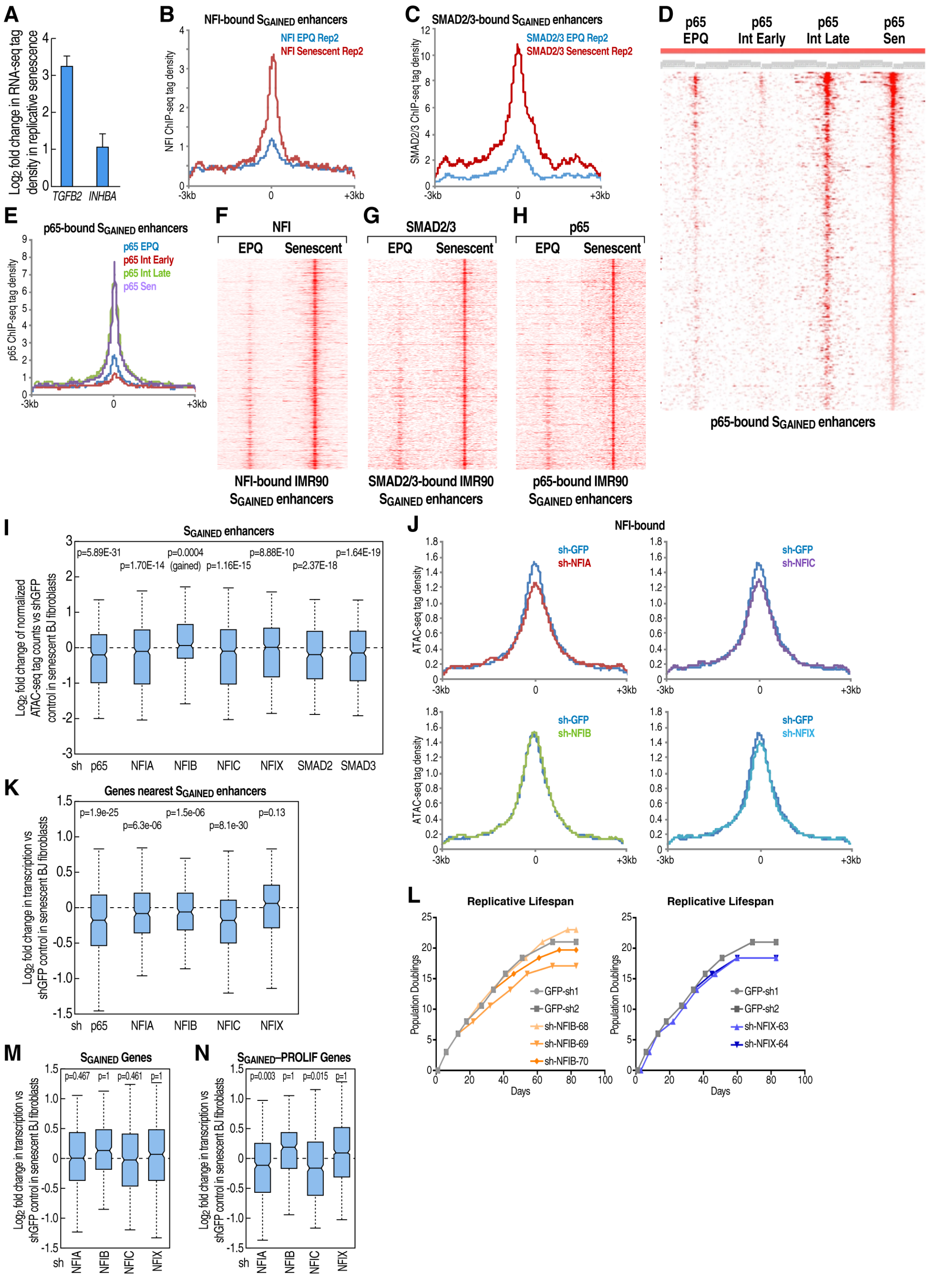

Suter et al. 2025, Fig. S3

Figure S3. NFI, p65, and SMAD2/3 activate SGAINED enhancers. **A)** Log_2 fold change in RNA-seq tag density for *TGFB2* and *INHBA* during replicative senescence. Error bars represent standard deviation (n=4 biological replicates). **B)** Meta-analysis of NFI tag density at the NFI-bound subset of SGAINED enhancers for NFI ChIP-seq replicate samples from senescent and EPQ BJ cells. **C)**

Meta-analysis of SMAD2/3 tag density at the SMAD2/3-bound subset of S_{GAINED} enhancers for SMAD2/3 ChIP-seq replicate samples from senescent and EPQ BJ cells. **D)** Heatmap of p65 ChIP-seq tag density at the p65-bound subset of S_{GAINED} enhancers for the indicated conditions. Each column displays a -/+ 3kb window from the center of p65 enhancer peaks. Enhancers were sorted based on p65 peak score in the senescent condition. **E)** Meta-analysis of p65 ChIP-seq tag density at the p65-bound subset of S_{GAINED} enhancers in the indicated conditions. **F-H)** ChIP-seq heatmaps for **F)** NFI, **G)** SMAD2/3, and **H)** p65 in EPQ or replicatively senescent IMR90 fibroblasts at enhancers gained in IMR90 replicative senescence. **I)** Box plot of change in ATAC-seq tag density at S_{GAINED} enhancers after 2-week shRNA-mediated knockdown of indicated transcription factor (TF) in senescent BJ fibroblasts. Fold change represents the ratio of normalized tag counts in each TF knockdown versus sh-GFP control. **J)** Meta-analysis of normalized ATAC-seq tag density at the NFI-bound subset of S_{GAINED} enhancers after 2 week-knockdown of the indicated NFI family member or GFP (control) in senescent BJ cells. **K)** Box plot of change in RNA-seq tag density of the expressed genes nearest to S_{GAINED} enhancers after 2-week knockdown of the indicated TF in senescent BJ fibroblasts. Fold change represents the ratio of normalized tag counts in each TF knockdown versus sh-GFP control. **L)** Population doublings over time of BJ fibroblasts after shRNA-mediated stable knockdown of NFIB (left), NFIX (right), or GFP (control). For each assay, NFI family members and GFP were targeted with more than one distinct shRNA construct in separate, parallel cultures. **M,N)** Box plots of change in RNA-seq tag density for **M)** 639 S_{GAINED} genes or **N)** 150 S_{GAINED}-PROLIF genes in indicated NFI family member stable knockdown versus sh-GFP control senescent BJ fibroblasts. Fold change represents the ratio of normalized tag counts in each NFI TF knockdown versus sh-GFP control. In these plots, p-values reflect the statistical significance of decreased tag density in each NFI TF knockdown relative to sh-GFP control.

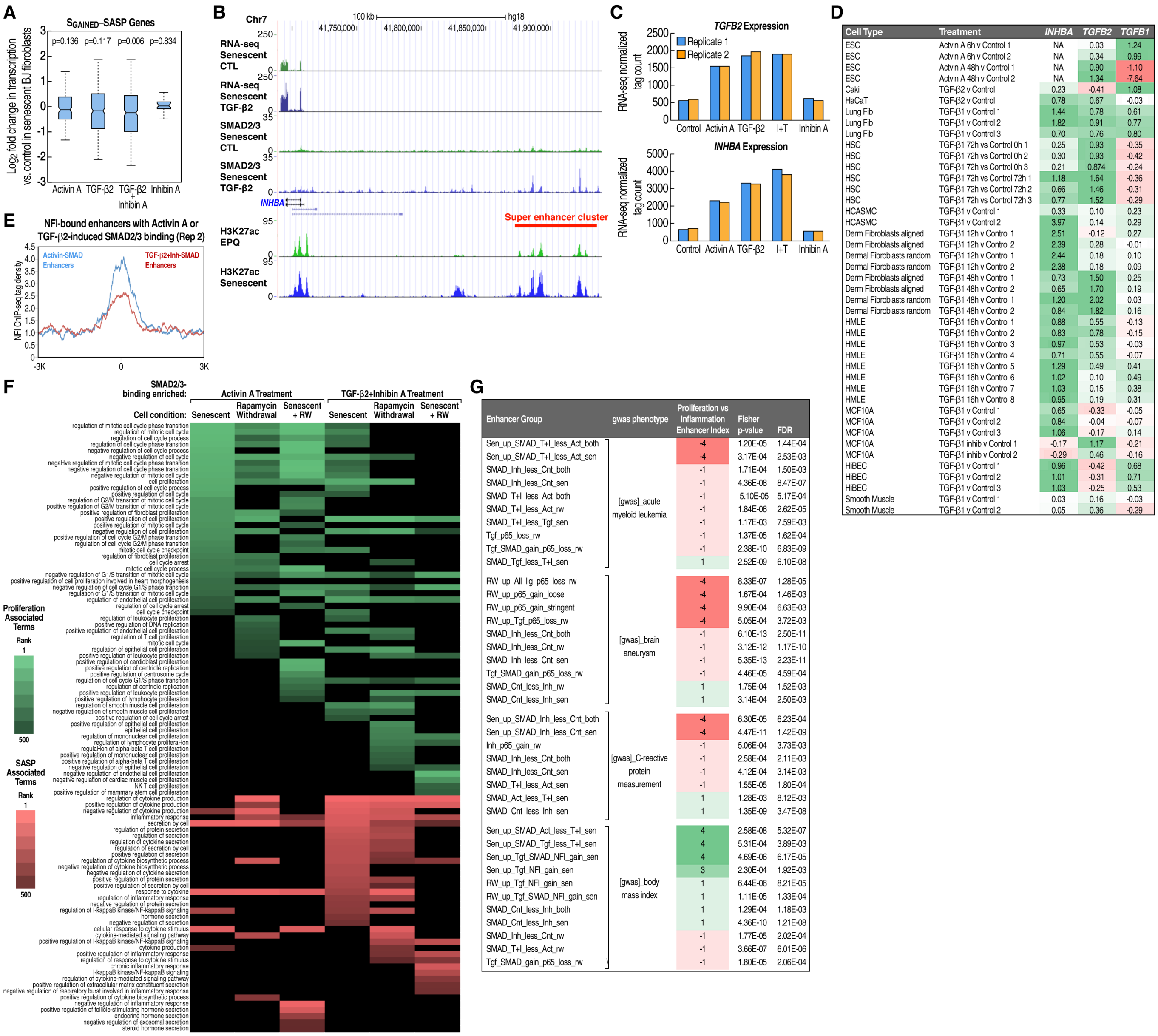

D

Cell Type	Treatment	*INHBA*	*TGFB2*	*TGFB1*
ESC	Actvin A 6h v Control 1	NA	0.03	1.24
ESC	Actvin A 6h v Control 2	NA	0.34	0.99
ESC	Actvin A 48h v Control 1	NA	0.90	-1.10
ESC	Actvin A 48h v Control 2	NA	1.34	-7.64
Caki	TGF-β2 v Control	0.23	-0.41	1.08
HaCaT	TGF-β2 v Control	0.78	0.67	-0.03
Lung Fib	TGF-β1 v Control 1	1.44	0.78	0.61
Lung Fib	TGF-β1 v Control 2	1.82	0.91	0.77
Lung Fib	TGF-β1 v Control 3	0.70	0.76	0.80
HSC	TGF-β1 72h vs Control 0h 1	0.25	0.93	-0.35
HSC	TGF-β1 72h vs Control 0h 2	0.30	0.93	-0.42
HSC	TGF-β1 72h vs Control 0h 3	0.21	0.874	-0.24
HSC	TGF-β1 72h vs Control 72h 1	1.18	1.64	-0.36
HSC	TGF-β1 72h vs Control 72h 2	0.66	1.46	-0.31
HSC	TGF-β1 72h vs Control 72h 3	0.77	1.52	-0.29
HCASMC	TGF-β1 v Control 1	0.33	0.10	0.23
HCASMC	TGF-β1 v Control 2	3.97	0.14	0.29
Derm Fibroblasts aligned	TGF-β1 12h v Control 1	2.51	-0.12	0.27
Derm Fibroblasts aligned	TGF-β1 12h v Control 2	2.39	0.28	-0.01
Dermal Fibroblasts random	TGF-β1 12h v Control 1	2.44	0.18	0.10
Dermal Fibroblasts random	TGF-β1 12h v Control 2	2.38	0.18	0.09
Derm Fibroblasts aligned	TGF-β1 48h v Control 1	0.73	1.50	0.25
Derm Fibroblasts aligned	TGF-β1 48h v Control 2	0.65	1.70	0.19
Dermal Fibroblasts random	TGF-β1 48h v Control 1	1.20	2.02	0.03
Dermal Fibroblasts random	TGF-β1 48h v Control 2	0.84	1.82	0.16
HMLE	TGF-β1 16h v Control 1	0.88	0.55	-0.13
HMLE	TGF-β1 16h v Control 2	0.83	0.78	-0.15
HMLE	TGF-β1 16h v Control 3	0.97	0.53	-0.03
HMLE	TGF-β1 16h v Control 4	0.71	0.55	-0.07
HMLE	TGF-β1 16h v Control 5	1.29	0.49	0.41
HMLE	TGF-β1 16h v Control 6	1.02	0.10	0.49
HMLE	TGF-β1 16h v Control 7	1.03	0.15	0.38
HMLE	TGF-β1 16h v Control 8	0.95	0.19	0.31
MCF10A	TGF-β1 v Control 1	0.65	-0.33	-0.05
MCF10A	TGF-β1 v Control 2	0.84	-0.04	-0.07
MCF10A	TGF-β1 v Control 3	1.06	-0.17	0.14
MCF10A	TGF-β1 inhib v Control 1	-0.17	1.17	-0.21
MCF10A	TGF-β1 inhib v Control 2	-0.29	0.46	-0.16
HiBEC	TGF-β1 v Control 1	0.96	-0.42	0.68
HiBEC	TGF-β1 v Control 2	1.01	-0.31	0.71
HiBEC	TGF-β1 v Control 3	1.03	-0.25	0.53
Smooth Muscle	TGF-β1 v Control 1	0.03	0.16	-0.03
Smooth Muscle	TGF-β1 v Control 2	0.05	0.36	-0.29

G

Enhancer Group	gwas phenotype	Proliferation vs Inflammation Enhancer Index	Fisher p-value	FDR
Sen_up_SMAD_T+I_less_Act_both	[gwas]_acute myeloid leukemia	-4	1.20E-05	1.44E-04
Sen_up_SMAD_T+I_less_Act_sen		-4	3.17E-04	2.53E-03
SMAD_Inh_less_Cnt_both		-1	1.71E-04	1.50E-03
SMAD_Inh_less_Cnt_sen		-1	4.36E-08	8.47E-07
SMAD_T+I_less_Act_both		-1	5.10E-05	5.17E-04
SMAD_T+I_less_Act_rw		-1	1.84E-06	2.62E-05
SMAD_T+I_less_Tgf_sen		-1	1.17E-03	7.59E-03
Tgf_p65_loss_rw		-1	1.37E-05	1.62E-04
Tgf_SMAD_gain_p65_loss_rw		-1	2.38E-10	6.83E-09
SMAD_Tgf_less_T+I_sen		1	2.52E-09	6.10E-08
RW_up_All_lig_p65_loss_rw	[gwas]_brain aneurysm	-4	8.33E-07	1.28E-05
RW_up_p65_gain_loose		-4	1.67E-04	1.46E-03
RW_up_p65_gain_stringent		-4	9.90E-04	6.63E-03
RW_up_Tgf_p65_loss_rw		-4	5.05E-04	3.72E-03
SMAD_Inh_less_Cnt_both		-1	6.10E-13	2.50E-11
SMAD_Inh_less_Cnt_rw		-1	3.12E-12	1.17E-10
SMAD_Inh_less_Cnt_sen		-1	5.35E-13	2.23E-11
Tgf_SMAD_gain_p65_loss_rw		-1	4.46E-05	4.59E-04
SMAD_Cnt_less_Inh_rw		1	1.75E-04	1.52E-03
SMAD_Cnt_less_Inh_sen		1	3.14E-04	2.50E-03
Sen_up_SMAD_Inh_less_Cnt_both	[gwas]_C-reactive protein measurement	-4	6.30E-05	6.23E-04
Sen_up_SMAD_Inh_less_Cnt_sen		-4	4.47E-11	1.42E-09
Inh_p65_gain_rw		-1	5.06E-04	3.73E-03
SMAD_Inh_less_Cnt_both		-1	2.58E-04	2.11E-03
SMAD_Inh_less_Cnt_sen		-1	4.12E-04	3.14E-03
SMAD_T+I_less_Act_sen		-1	1.55E-05	1.80E-04
SMAD_Act_less_T+I_sen		1	1.28E-03	8.12E-03
SMAD_Cnt_less_Inh_sen		1	1.35E-09	3.47E-08
Sen_up_SMAD_Act_less_T+I_sen	[gwas]_body mass index	4	2.58E-08	5.32E-07
Sen_up_SMAD_Tgf_less_T+I_sen		4	5.31E-04	3.89E-03
Sen_up_Tgf_SMAD_NFI_gain_sen		4	4.69E-06	6.17E-05
Sen_up_Tgf_NFI_gain_sen		3	2.30E-04	1.92E-03
RW_up_Tgf_NFI_gain_sen		1	6.44E-06	8.21E-05
RW_up_Tgf_SMAD_NFI_gain_sen		1	1.11E-05	1.33E-04
SMAD_Cnt_less_Inh_both		1	1.29E-04	1.18E-03
SMAD_Cnt_less_Inh_sen		1	4.36E-10	1.21E-08
SMAD_Inh_less_Cnt_rw		-1	1.77E-05	2.02E-04
SMAD_T+I_less_Act_rw		-1	3.66E-07	6.01E-06
Tgf_SMAD_gain_p65_loss_rw		-1	1.80E-05	2.06E-04

Figure S4. Distinct roles of TGFβ ligands Activin A and TGF-β2 in regulating replicative senescence enhancer programs. **A)** Box plots of change in RNA-seq tag density of the 150 SGAINED-SASP gene subset in senescent BJ fibroblasts treated with Activin A, TGF-β2, TGF-β2+Inhibin A, or Inhibin A versus vehicle control. **B)** UCSC genome browser tracks of indicated ChIP-seq and RNA-seq profiles at the *TGFB2* gene locus. **C)** Normalized RNA-seq tag counts for *TGFB2* and *INHBA* genes in replicatively senescent BJ fibroblasts treated for two weeks with vehicle control (DMSO) or indicated SMAD2/3-regulating ligands (I+T, Inhibin A + TGF-β2). **D)** Log_2 fold change of RNA-seq tag density in the gene bodies of *INHBA*, *TGFB2*, or *TGFB1* for different cell types treated with Activin A, TGF-β2, or TGF-β1. Raw data were obtained from publicly available RNA-seq datasets (see Extended methods, *SI Appendix*). **E)** Meta-analysis of normalized NFI ChIP-seq tag density for replicate samples from replicatively senescent BJ fibroblasts showing NFI recruitment to Activin A or TGF-β2+Inhibin A treatment-specific

SMAD2/3-bound enhancers (normalized to DMSO control distribution). Plots were centered on enhancers with >2-fold increase in SMAD2/3 ChIP-seq tag density in both senescent and rapamycin-withdrawn HPGA BJ fibroblasts. **F)** Heatmap of GO-Biology terms associated with either regulation of proliferation (green) or SASP (red) identified in GREAT analysis of SMAD2/3-bound enhancer sets from listed conditions. All terms associated with either category were extracted from the top 500 GO-Biology output for each enhancer set, rank normalized, and colorized based on the rank and category of association. All terms present in any of the 6 enhancer sets are presented, with black colorization indicating that a term was not in the top 500 for a given enhancer set (see Extended methods, *SI Appendix*). **G)** GWAS age-related traits associated with either proliferation or SASP enhancer sets. 83 enhancer sets identified in the project were scored based on their association with either program (negative values, colored in red, represent an association with SASP/inflammation, whereas positive values, colored in green, indicate an association with proliferation control). These enhancer sets were tested for enrichment of SNPs linked to 531 GWAS/PheWAS traits. Selected traits with enrichment of GWAS/PheWAS SNPs in enhancer sets that were most strongly associated with either regulation of proliferation or SASP/inflammation are shown (FDR < 0.05; see Extended methods, *SI Appendix*).

Table S1. Gene cluster analysis for up-/down-regulated genes in replicatively senescent BJ fibroblasts and rapamycin withdrawal (RW)-gained genes in HPGA cells 2 weeks after treatment cessation, based on combined RNA-seq and GRO-seq data.

	max gap	% clustered	% clustered for random background	most frequent cluster size (% of clustered genes)	largest cluster size (% of clustered genes)	p-value
639 UP-reg genes					*	
	0	21.64	5.07	2 (15.2%)	3 (3.64%)	< 1.0E-10
	1	29.82	9.94	2 (19.49%)	5 (1.01%)	< 1.0E-10
	2	35.87	14.5	2 (23.39%)	5 (2.02%)	< 1.0E-10
598 DOWN-reg genes						
	0	9.64	5.58	2 (8.93%)	4 (0.71%)	1.39E-03
	1	17.86	10.83	2 (16.07%)	4 (0.71%)	5.53E-05
	2	22.14	15.79	2 (18.21%)	4 (0.71%)	9.64E-04
252 RW-Gained genes						
	0	12.11	2.17	2 (10.76%)	3 (1.35%)	< 1.0E-10
	1	17.49	4.41	2 (16.14%)	3 (1.35%)	< 1.0E-10
	2	19.28	6.48	2 (17.94%)	3 (1.35%)	1.02E-08
650 random BJ-expressed genes						
	0	5.74	5.67	2 (5.22%)	3 (0.52%)	4.80E-01
	1	11.48	11.07	2 (9.39%)	3 (2.09%)	4.08E-01
	2	15.3	16.11	2 (12.17%)	3 (3.13%)	3.50E-01

* This analysis alone was performed by excluding a single cluster of 15 consecutive PCDH genes in the up-regulated gene set. Exclusion of the PCDH gene cluster did not affect overall p-values and minimally altered other results, indicating that it was not a biasing feature of the data set.

Table S2. Paired enhancer sets used in GREAT meta-analysis**.** Each square of the table notes a set of enhancers showing greater SMAD2/3 ChIP-seq tag density in the first (**bolded**) ligand treatment relative to the second (**bolded**) ligand treatment of BJ fibroblasts in the indicated (*italicized*) condition. Each row consists of a pair of such enhancer sets with an inverse SMAD2/3 ChIP-seq binding ratio. These enhancer sets were compared in a pairwise manner in the GREAT meta-analysis (see Methods in Main Text and Extended methods in *SI Appendix*). The right-side column consists of enhancer sets where the numerator condition involved treatment with Inhibin A (an Activin A inhibitor), whereas the left-side column consists of the inverse condition (uninhibited Activin A activity). SASP: Senescence Associated Secretory Phenotype; HPGA: High Passage Growth Arrested.

**Proliferation-Associated Enhancer Sets**	**Paired SASP-Associated Enhancer Sets**
Higher SMAD2/3 binding in **Activin A** vs **TGF-β2+Inhibin A** treatment in *senescent* cells	Higher SMAD2/3 binding in **TGF-β2+Inhibin A** vs **Activin A** treatment in *senescent* cells
Higher SMAD2/3 binding in **Activin A vs TGF-β2+Inhibin A** treatment in *rapamycin withdrawn HGPA* cells	Higher SMAD2/3 binding in **TGF-β2+Inhibin A** vs **Activin A** treatment in *rapamycin withdrawn HGPA* cells
Higher SMAD2/3 binding in **Activin A** vs **TGF-β2+Inhibin A** treatment in both *senescent and rapamycin withdrawn HGPA* cells	Higher SMAD2/3 binding in **TGF-β2+Inhibin A** vs **Activin A** treatment in both *senescent and rapamycin withdrawn* HGPA cells
Higher SMAD2/3 binding in both **Activin A** vs **TGF-β2+Inhibin A** treatment and **TGF-β2** vs **TGF-β2+Inhibin A** treatment in *senescent* cells	Higher SMAD2/3 binding in both **TGF-β2+Inhibin A** vs **Activin A** treatment and **TGF-β2** vs **TGF-β2+Inhibin A** treatment in *senescent* cells
Higher SMAD2/3 binding in both **Activin A** vs **TGF-β2+Inhibin A** treatment and **TGF-β2** vs **TGF-β2+Inhibin A** treatment in *rapamycin withdrawn HGPA* cells	Higher SMAD2/3 binding in both **TGF-β2+Inhibin A** vs **Activin A** treatment and **TGF-β2** vs **TGF-β2+Inhibin A** treatment in *rapamycin withdrawn HGPA* cells
Higher SMAD2/3 binding in **TGF-β2** vs **TGF-β2+Inhibin A** treatment in *senescent* cells	Higher SMAD2/3 binding in **TGF-β2+Inhibin A** vs **TGF-β2** treatment in *senescent* cells
Higher SMAD2/3 binding in **TGF-β2** vs **TGF-β2+Inhibin A** treatment in *rapamycin withdrawn HGPA* cells	Higher SMAD2/3 binding in **TGF-β2+Inhibin A** vs **TGF-β2** treatment in *rapamycin withdrawn HGPA* cells
Higher SMAD2/3 binding in **TGF-β2** vs **TGF-β2+Inhibin A** treatment in both *senescent and rapamycin withdrawn HGPA* cells	Higher SMAD2/3 binding in **TGF-β2+Inhibin A** vs **TGF-β2** treatment in both *senescent and rapamycin withdrawn HGPA* cells
Higher SMAD2/3 binding in **Control** vs **Inhibin A** treatment in *senescent* cells	Higher SMAD2/3 binding in **Inhibin A** vs **Control** treatment in *senescent* cells
Higher SMAD2/3 binding in **Control** vs **Inhibin A** treatment in *rapamycin withdrawn HGPA* cells	Higher SMAD2/3 binding in **Inhibin A** vs **Control** treatment in *rapamycin withdrawn HGPA* cells
Higher SMAD2/3 binding in **Control** vs **Inhibin A** treatment in both *senescent and rapamycin withdrawn HGPA* cells	Higher SMAD2/3 binding in **Inhibin A** vs **Control** treatment in both *senescent and rapamycin withdrawn HGPA* cells

Table S3. Oligos for constructing shRNA lentiviral vectors.

Enhancer-proximal gene	Enhancer	shRNA target	Oligo strand	Oligo Sequence	Design Algorithm
ID1	1	GCTTTGTGATCTGAAACAAGT	Forward	CCGGGCTTTGTGATCTGAAACAAGTCTCGAGACTTGTTTCAGATCACAAAGCTTTTTG	DSIR
ID1	1	GCTTTGTGATCTGAAACAAGT	Reverse	AATTCAAAAAGCTTTGTGATCTGAAACAAGTCTCGAGACTTGTTTCAGATCACAAAGC	DSIR
IGFBP5	1	GGAGCTGGAAAGTACTGAAGC	Forward	CCGGGGAGCTGGAAAGTACTGAAGCCTCGAGGCTTCAGTACTTTCCAGCTCCTTTTTG	DSIR
IGFBP5	1	GGAGCTGGAAAGTACTGAAGC	Reverse	AATTCAAAAAGGAGCTGGAAAGTACTGAAGCCTCGAGGCTTCAGTACTTTCCAGCTCC	DSIR
INHBA	1	GAGATTAGTGTAAAGGAAA	Forward	CCGGGAGATTAGTGTAAAGGAAATTCAAGAGATTTCCTTTACACTAATCTCTTTTTG	Dharmacon
INHBA	1	GAGATTAGTGTAAAGGAAA	Reverse	AATTCAAAAAGAGATTAGTGTAAAGGAAATCTCTTGAATTTCCTTTACACTAATCTC	Dharmacon
INHBA	2	GAGCCAAACTAGCACCAAA	Forward	CCGGGAGCCAAACTAGCACCAAATTCAAGAGATTTGGTGCTAGTTTGGCTCTTTTTG	Dharmacon
INHBA	2	GAGCCAAACTAGCACCAAA	Reverse	AATTCAAAAAGAGCCAAACTAGCACCAAATCTCTTGAATTTGGTGCTAGTTTGGCTC	Dharmacon
CD36	1	GGTACAAAGTTATTCTTGATT	Forward	CCGGGGTACAAAGTTATTCTTGATTCTCGAGAATCAAGAATAACTTTGTACCTTTTTG	DSIR
CD36	1	GGTACAAAGTTATTCTTGATT	Reverse	AATTCAAAAAGGTACAAAGTTATTCTTGATTCTCGAGAATCAAGAATAACTTTGTACC	DSIR
CD36	2	GCTAAATAGTATTCCACAATG	Forward	CCGGGCTAAATAGTATTCCACAATGCTCGAGCATTGTGGAATACTATTTAGCTTTTTG	DSIR
CD36	2	GCTAAATAGTATTCCACAATG	Reverse	AATTCAAAAAGCTAAATAGTATTCCACAATGCTCGAGCATTGTGGAATACTATTTAGC	DSIR
Gene target	shRNA Number				
p65	1	GCCTTAATAGTAGGGTAAGTT	Forward	CCGGGCCTTAATAGTAGGGTAAGTTCTCGAGAACTTACCCTACTATTAAGGCTTTTTG	TRC
p65	1	GCCTTAATAGTAGGGTAAGTT	Reverse	AATTCAAAAAGCCTTAATAGTAGGGTAAGTTCTCGAGAACTTACCCTACTATTAAGGC	TRC
p65	2	CGGATTGAGGAGAAACGTAAA	Forward	CCGGCGGATTGAGGAGAAACGTAAACTCGAGTTTACGTTTCTCCTCAATCCGTTTTTG	TRC
p65	2	CGGATTGAGGAGAAACGTAAA	Reverse	AATTCAAAAACGGATTGAGGAGAAACGTAAACTCGAGTTTACGTTTCTCCTCAATCCG	TRC

Table S4. Acquired shRNA constructs used in this study.

**gene-targeting shRNA constructs**				
Gene Symbol	Library	Catalog Number	Sense strand sequence	shRNA clone identifier used in figures
NFIA	Hs_TRC	RHS3979-9583917	GCGCAGTTACAACTTCACTAT	17
NFIA	Hs_TRC	RHS3979-9583920	CTGTTAAGGAACTCGATTTAT	20
NFIA	Hs_TRC	RHS3979-9583921	GAAGTCGGAGAAGTCTGGTTT	21
NFIB	Hs_TRC	RHS3979-9582369	CCGTGCTGTGTCTTATCCAAT	69
NFIB	Hs_TRC	RHS3979-9582368	CCGTTGCCATTTCCAACACAA	68
NFIB	Hs_TRC	RHS3979-9582370	GCACGAAAGAGATCAAGATAT	70
NFIC	Hs_TRC	RHS3979-9582400	CCCGGTGAAGAAGACAGAGAT	100
NFIC	Hs_TRC	RHS3979-9582397	CCACCCAAGCAAGAAGACAAA	97
NFIC	Hs_TRC	RHS3979-9582399	GCGGCACAAATCGGGCTCGAT	99
NFIC	Hs_TRC	RHS3979-9582401	CCTCCGCTCTGCATTTCCCTA	101
NFIX	Hs_TRC	RHS3979-9582463	CCTGTTGATGACGTGTTCTAT	63
NFIX	Hs_TRC	RHS3979-9582464	ACTGGATCTTTATCTGGCTTA	64
SMAD2	Hs_TRC	RHS3979-9607426	CCTAAGTGATAGTGCAATCTT	
SMAD3	Hs_TRC	RHS3979-9587417	CTGTGTGAGTTCGCCTTCAAT	
**shRNA Controls**				
Gene Symbol	Library	Item	Sense Sequence	
GFP	TRC	TRC GFP control	TACAACAGCCACAACGTCTAT	
(None)	GIPZ	GIPZ non-targeting	CTCGCTTGGGCGAGAGTAA	

Table S5. Primers used in this study.

Primer Name	Primer Sequence
qPCR Primers	
TS_CD36mRNA_F	ggctgcaggtcaacctattg
TS_CD36mRNA_R	ccaatggtcccagtctcatt
TS_IGFBP5mRNA_F	tgcacctgagatgagacagg
TS_IGFBP5mRNA_R	gaatcctttgcggtcacaat
TS_ID1mRNA_F	aaacgtgctgctctacgaca
TS_ID1mRNA_R	tagtcgatgacgtgctggag
TS_INHBAmRNA_F1	ggagggcagaaatgaatgaa
TS_INHBAmRNA_R1	ccttggaaatctcgaagtgc
GRO-seq Sequencing Primers	
oNTI200	CAAGCAGAAGACGGCATA
oNTI200-TruSeqID1:	CAAGCAGAAGACGGCATACGAGAT CGTGAT GTGACTGGAGTT CAGACGTGTGCTCTTCCGATC
oNTI200-TruSeqID2:	CAAGCAGAAGACGGCATACGAGAT ACATCG GTGACTGGAGTT CAGACGTGTGCTCTTCCGATC
oNTI200-TruSeqID3:	CAAGCAGAAGACGGCATACGAGAT GCCTAA GTGACTGGAGTT CAGACGTGTGCTCTTCCGATC
oNTI200-TruSeqID4:	CAAGCAGAAGACGGCATACGAGAT TGGTCA GTGACTGGAGTT CAGACGTGTGCTCTTCCGATC
oNTI200-TruSeqID5:	CAAGCAGAAGACGGCATACGAGAT CACTGT GTGACTGGAGTT CAGACGTGTGCTCTTCCGATC
oNTI200-TruSeqID6:	CAAGCAGAAGACGGCATACGAGAT ATTGGC GTGACTGGAGTT CAGACGTGTGCTCTTCCGATC
oNTI201	AATGATACGGCGACCACCGACAGGTTCAGAGTTCTACAGTCCGACG
Illumina small RNA-seq primer	CGACAGGTTCAGAGTTCTACAGTCCGACGATC
oNTI223	pGATCGTCGGACTGTAGAACTCT;CAAGCAGAAGACGGCATACGATTTTTTTTTTTTTTTTTTTVN
	p = 5′ phosphorylation
	";" = abasic dSpacer furan
	VN = degenerate nucleotides

SI References

1. W. Li, *et al.*, Functional roles of enhancer RNAs for oestrogen-dependent transcriptional activation. *Nature* **498**, 516–520 (2013).

2. S. Heinz, *et al.*, Simple combinations of lineage-determining transcription factors prime cis-regulatory elements required for macrophage and B cell identities. *Mol. Cell* **38**, 576–589 (2010).

3. Y. Zhou, *et al.*, Metascape provides a biologist-oriented resource for the analysis of systems-level datasets. *Nat. Commun.* **10**, 1523 (2019).

4. A. Subramanian, *et al.*, Gene set enrichment analysis: A knowledge-based approach for interpreting genome-wide expression profiles. *Proc. Natl. Acad. Sci.* **102**, 15545–15550 (2005).

5. V. K. Mootha, *et al.*, PGC-1α-responsive genes involved in oxidative phosphorylation are coordinately downregulated in human diabetes. *Nat. Genet.* **34**, 267–273 (2003).

6. S. S. P. Rao, *et al.*, A 3D map of the human genome at kilobase resolution reveals principles of chromatin looping. *Cell* **159**, 1665–1680 (2014).

7. M. Imakaev, *et al.*, Iterative correction of Hi-C data reveals hallmarks of chromosome organization. *Nat. Methods* **9**, 999–1003 (2012).

8. F. Jin, *et al.*, A high-resolution map of the three-dimensional chromatin interactome in human cells. *Nature* **503**, 290–294 (2013).

9. Y. Benjamini, Y. Hochberg, Controlling the False Discovery Rate: A Practical and Powerful Approach to Multiple Testing. *J. R. Stat. Soc. Ser. B Methodol.* **57**, 289–300 (1995).

10. F. Pazos Obregón, *et al.*, Cluster Locator, online analysis and visualization of gene clustering. *Bioinforma. Oxf. Engl.* **34**, 3377–3379 (2018).

11. S. C. Johnson, X. Dong, J. Vijg, Y. Suh, Genetic evidence for common pathways in human age-related diseases. *Aging Cell* **14**, 809–817 (2015).

12. S. C. Johnson, *et al.*, Network analysis of mitonuclear GWAS reveals functional networks and tissue expression profiles of disease-associated genes. *Hum. Genet.* **136**, 55–65 (2017).
